# Radiation and Dose-densification of R-CHOP in Primary Mediastinal B-cell Lymphoma: Subgroup Analysis of the UNFOLDER Trial

**DOI:** 10.1097/HS9.0000000000000917

**Published:** 2023-07-05

**Authors:** Gerhard Held, Lorenz Thurner, Viola Poeschel, German Ott, Christian Schmidt, Konstantinos Christofyllakis, Andreas Viardot, Peter Borchmann, Walburga Engel-Riedel, Norbert Frickhofen, Maike Nickelsen, Ofer Shpilberg, Mathias Witzens-Harig, Frank Griesinger, Beate Krammer-Steiner, Andreas Neubauer, Peter de Nully Brown, Massimo Federico, Bertram Glass, Norbert Schmitz, Gerald Wulf, Lorenz Truemper, Moritz Bewarder, Niels Murawski, Stephan Stilgenbauer, Andreas Rosenwald, Bettina Altmann, Marianne Engelhard, Heinz Schmidberger, Jochen Fleckenstein, Christian Berdel, Markus Loeffler, Marita Ziepert

**Affiliations:** 1 Department of Internal Medicine 1, Westpfalz-Klinikum, Kaiserslautern, Germany; 2 Department of Internal Medicine 1 (Oncology, Hematology, Clinical Immunology and Rheumatology), Saarland University Medical School, Homburg/Saar, Germany; 3 Department of Clinical Pathology, Robert-Bosch-Krankenhaus and Dr. Margarete Fischer-Bosch Institute of Clinical Pharmacology, Stuttgart, Germany; 4 Department of Medicine III, University Hospital, Munich, Germany; 5 Department of Internal Medicine III, University Hospital Ulm, Germany; 6 Department of Hematology and Oncology, University Hospital of Cologne, Germany; 7 Pulmonary Clinic, Krankenhaus Köln-Merheim, Germany; 8 Department of Internal Medicine III, Helios Dr.Horst-Schmidt-Kliniken, Wiesbaden, Germany; 9 Oncology Lerchenfeld, Hamburg, Germany; 10 Department of Hematology, Rabin Medical Center, Beilinson Hospital, Petah-Tiqwa, Israel; 11 Department of Internal Medicine V, University of Heidelberg, Germany; 12 Department of Internal Oncology, Pius-Hospital, Oldenburg, Germany; 13 Department of Internal Medicine, Klinikum Rostock Südstadt, Germany; 14 Department of Hematology, Oncology and Immunology, University Hospital Marburg, Germany; 15 Department of Hematology, Rigshospitalet, Copenhagen, Denmark; 16 CHIMOMO Department, University of Modena and Reggio Emilia, Italy; 17 Department of Hematology and Stem Cell Transplantation, Helios Klinikum Berlin-Buch, Germany; 18 Department of Medicine A, Hematology, Oncology and Pneumology, University Hospital Münster, Germany; 19 Department of Hematology and Oncology, Georg August University of Goettingen, Germany; 20 Institute of Pathology, University of Wuerzburg, and Comprehensive Cancer Center Mainfranken, Germany; 21 Institute for Medical Informatics, Statistics and Epidemiology, University Leipzig, Germany; 22 Department of Radiotherapy, University Hospital Essen, Germany; 23 Department of Radiooncology and Radiotherapy, University Medical Center, Mainz, Germany; 24 Department of Radiotherapy and Radiation Oncology, Saarland University Medical School, Homburg/Saar, Germany

## Abstract

UNFOLDER (NCT00278408, EUDRACT 2005-005218-19) is a phase-3 trial in patients with aggressive B-cell lymphoma and intermediate prognosis, including primary mediastinal B-cell lymphoma (PMBCL). In a 2 × 2 factorial design, patients were randomized to 6× R-CHOP-14 or R-CHOP-21 (rituximab, cyclophosphamide, doxorubicin, vincristine, and prediso(lo)ne) and to consolidation radiotherapy to extralymphatic/bulky disease or observation. Response was assessed according to the standardized criteria from 1999, which did not include F-18 fluordesoxyglucose positron emission tomography/computed tomography (FDG-PET) scans. Primary end point was event-free survival (EFS). A subgroup of 131 patients with PMBCLs was included (median age, 34 y; 54% female, 79% elevated lactate dehydrogenase (LDH), 20% LDH >2× upper limit of normal [ULN], and 24% extralymphatic involvement). Eighty-two (R-CHOP-21: 43 and R-CHOP-14: 39) patients were assigned to radiotherapy and 49 (R-CHOP-21: 27, R-CHOP-14: 22) to observation. The 3-year EFS was superior in radiotherapy arm (94% [95% confidence interval (CI), 89-99] versus 78% [95% CI, 66-89]; *P* = 0.0069), due to a lower rate of partial responses (PRs) (2% versus 10%). PR triggered additional treatment, mostly radiotherapy (n = 5; PR: 4; complete response/unconfirmed complete response: 1). No significant differences were observed in progression-free survival (PFS) (95% [95% CI, 90-100] versus 90% [95% CI, 81-98]; *P* = 0.25) nor in overall survival (OS) (98% [95% CI, 94-100] versus 96% [95% CI, 90-100]; *P* = 0.64). Comparing R-CHOP-14 and R-CHOP-21, EFS, PFS, and OS were not different. A prognostic marker for adverse outcome was elevated LDH >2× ULN (EFS: *P* = 0.016; PFS: *P* = 0.0049; OS: *P* = 0.0014). With the limitation of a pre-PET-era trial, the results suggest a benefit of radiotherapy only for patients responding to R-CHOP with PR. PMBCL treated with R-CHOP have a favorable prognosis with a 3-year OS of 97%.

## Graphical abstract

**Figure:**
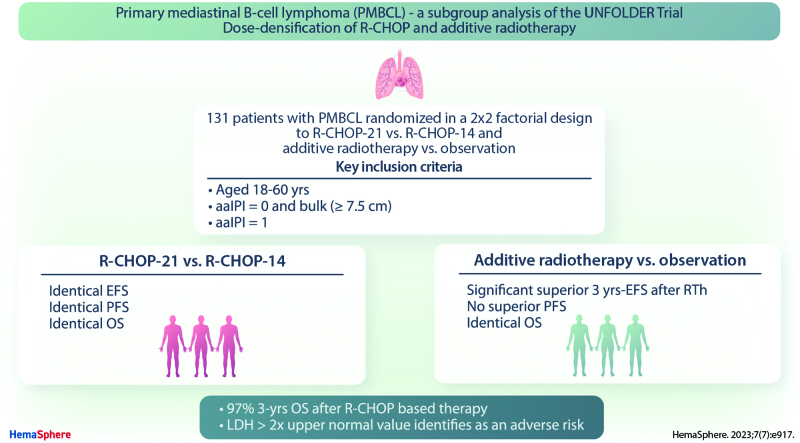

## INTRODUCTION

Primary mediastinal B-cell lymphoma (PMBCL) is a distinct entity of aggressive lymphoma, which typically presents as a bulky anterior mediastinal mass in young patients with a female predominance.^1^ PMBCL cells typically lack expression of sIg, but commonly show a weak co-expression of CD30. Biologically activated NF-kB- and JAK2-pathways, for example, by gain of 9p24 are a hallmark of PMBCL together with frequent loss of functional HLA-II complexes.^2^ It has been described that these differences in biology compared with classic, not otherwise specified diffuse large B-cell lymphoma (DLBCL NOS) is also accompanied by a different, more favorable prognosis with less common late relapses. Chemotherapy is based on the CHOP (cyclophosphamide, doxorubicin, vincristine, and prednisone) or more intensive regimens such as MACOP-B (methotrexate with leucovorin rescue, doxorubicin, cyclophosphamide, vincristine, prednisone, and bleomycin), with a major benefit due to addition of immunotherapy with rituximab (R).^3,4^ A potential benefit of densification by a biweekly application of R-CHOP was raised by an exploratory analysis of 50 patients with PMBCL included in the phase-III UK NCRI R-CHOP-14 versus -21 trial, where a trend to a better progression-free survival (PFS) and overall survival (OS) was detected after R-CHOP-14.^5^

Given the favorable course described for PMBCL and the potential long-term side effects of mediastinal radiotherapy, its role is also controversial. A very good outcome has been reported in a phase-II trial for an intensified, modified CHOP-like regimen with dose-adjusted EPOCH-R (dose-adjusted treatment: etoposide, prednisone, vincristine, cyclophosphamide, doxorubicin, and rituximab) alone.^6^ Moreover, since the conception of the UNFOLDER trial the diagnostic standards changed - nowadays F-18 fluordesoxyglucose positron emission tomography/computed tomography (FDG-PET/CT) is included for initial and final staging. Also potential therapeutic options for relapsed/refractory (r/r) PMBCL have increased for instance by promising results of the combination of brentuximab vedotin and nivolumab, and particularly of chimeric antigen receptor (CAR) T-cells.^7–12^

Here, we analyzed 131 patients with PMBCL treated within the prospective randomized phase-III UNFOLDER trial. Patients with bulky or extralymphatic disease were randomized in a 2 × 2 factorial design to receive either 6× R-CHOP-21 versus 6× R-CHOP-14 (chemotherapy comparison) and to radiotherapy to bulk or extralymphatic sites versus observation (radiotherapy comparison).

## Methods

### Patients

The evaluation reported here is a planned subgroup analysis encompassing the PMBCL patients within the UNFOLDER trial. The UNFOLDER trial is a 2 × 2 factorial design, international, phase-III trial from 148 clinical sites in Denmark, Israel, Italy, and Germany. It was coordinated by the German High-grade Non-Hodgkin’s Lymphoma Study Group, which is now part of the German Lymphoma Alliance. The study was conducted in accordance with the Helsinki declaration. The protocol and its amendments were approved by the ethics committee of each participating center. Additional information about trial oversight and amendments is provided in the Suppl. Tables S1-S2.

Patients between 18 and 60 years of age were eligible for randomization if they presented with previously untreated aggressive B-cell lymphoma according to the World Health Organization (WHO) classification (3rd edition, 2001 and 4th edition, 2008) and if they had 1 risk factor according to the age-adjusted International Prognostic Index (aaIPI) (lactate dehydrogenase [LDH] above the upper limit of normal [ULN], Eastern Cooperative Oncology Group [ECOG] performance status 2 or 3, or Ann Arbor stage III or IV), or no risk factor according to aaIPI but bulky disease (diameter of single or conglomerate tumor ≥7.5 cm). Patients with central nervous system (CNS) involvement were excluded. A complete list of exclusion criteria are provided in the protocol in the appendix.

### Treatments

R-CHOP comprised rituximab (375 mg/m^2^), cyclophosphamide (750 mg/m^2^), doxorubicin (50 mg/m^2^), vincristine (1.4 mg/m^2^ with a maximum total dose of 2 mg) administered on day 1, plus oral prednisone/prednisolone (100 mg) administered on days 1–5. R-CHOP-14 was repeated every 2 weeks with mandatory granulocyte colony-stimulating factor (G-CSF)-support, R-CHOP-21 every 3 weeks.

Patients qualifying for radiotherapy had bulky disease (≥7.5 cm) not surgically removed and/or extralymphatic involvement amenable for radiotherapy. Involvements of the localizations were planned not to receive radiotherapy: bone marrow, lung, liver, kidney, small intestine, colon, ascites, pericardial, and pleural effusions. Radiotherapy was administered at a total dose of 39.6 Gy involved-field with 1.8 Gy/fraction 5 times a week. Radiotherapy should be started 2–6 weeks after the last cycle of chemotherapy. A radiotherapy review panel performed quality control. Initial staging images the radiotherapy plan and verification images evaluated.

### Response assessment and end points

Response was assessed according to the International Workshop to Standardize Response Criteria for Non-Hodgkin’s Lymphomas published in 1999.^13^ Responding patients with residual masses were assessed as unconfirmed complete response (CRu) (residual lymphoma regressed by >75% in the sum of the product of greatest diameters [SPD]) or partial response (PR) (residual lymphoma regressed by ≥50% in SPD). PR also indicated the need for additional treatment indicated by vital lymphoma in biopsy or by the judgement of the investigator. Final response was assessed 2 weeks after the start of sixth cycle of R-CHOP in the observation arm. Patients with CRu/PR as demonstrated by restaging after completion of immuno-chemotherapy received a confirmation of remission 4 weeks thereafter. Final response was assessed after end of radiotherapy simultaneously with first follow-up in the radiotherapy arm. First follow-up examination was done 3 months after the restaging after 6 cycles of R-CHOP.

Event-free survival (EFS) was the primary end point, defined as the time from randomization until one of the following events had occurred: progression during therapy, no change, termination of therapy due to toxicity without complete response (CR)/CRu, no CR/CRu at the end of study treatment, relapse after CR/CRu, death from any cause, or application of additional treatment, whichever came first. Radiotherapy as additional treatment was not counted as an event in patients of the observation arm who received radiotherapy due to the results of the interim analysis. In these patients, response assessment was performed after radiotherapy. Key secondary end points were PFS, defined as the date from randomization to disease progression, relapse, or any cause of death and OS defined as the time from randomization to death of any cause.

Other secondary end points were rate of CRs and progressive disease, relapse patterns (relapse in regions treated with radiotherapy, relapse in primarily involved regions, and in not primarily involved regions), safety (adverse events, serious adverse events, rate of secondary neoplasia, selected laboratory parameters, including leucocytes, thrombocytes, and hemoglobin), adherence to protocol (duration of cycles, cumulative dose, and dose intensity), and health-economic aspects (using the cumulative dose of chemotherapy drugs and rituximab).

For patients qualifying for radiotherapy, an as-treated analysis was performed for PFS and OS. Patients who were randomized in observation arms, but received radiotherapy were analyzed in radiotherapy arms.

### Statistical analysis

Here we report a planned subgroup analysis of patients presenting with PMBCL within the UNFOLDER trial. The UNFOLDER trial was planned for patients qualifying for radiotherapy in a 2 × 2 factorial design to show potential differences in comparison of chemotherapy dose-densification (6× R-CHOP-14 versus 6× R-CHOP-21) and in the impact of radiotherapy application to bulky disease and/or extralymphatic involvement (6× R-CHOP-21/14 with radiotherapy versus 6× R-CHOP-21/14 observation).

Randomization was done before the start of R-CHOP using the Pocock minimization algorithm with a random component after stratification for centers, serum lactate dehydrogenase (normal versus elevated), stage (Ann Arbor stage I, II versus III, IV), ECOG performance status (0.1 versus 2.3), bulky disease (no versus yes), and extralymphatic sites (no versus yes). Randomization was performed at a ratio of 1:1:1:1 in the following treatment arms: 6× R-CHOP–21+ radiotherapy, or 6× R-CHOP-14+ radiotherapy or only 6× R-CHOP–21 or 6× R-CHOP-14. In addition, patients not qualifying for consolidation radiotherapy were randomized at a ratio of 1:1 to receive either 6× R-CHOP–21 or 6× R-CHOP-14. The complete trial was powered to show a hazard ratio (HR) of 0.615 or an improvement of 10% in the primary end point of 3-year EFS (71%–81%) for dose-densification and for radiotherapy. For more details, we refer to the separate publication of the UNFOLDER trial.^14^

A planned interim analysis was performed on July 1, 2012. In total 443 patients were evaluable for analysis, of whom 285 were qualified and randomized to receive radiotherapy. In this analysis, the predefined formal criterion of discontinuation was fulfilled, because EFS of the 139 patients randomized to receive radiotherapy was significantly better compared with those randomized into the observational arm, with a *P*-value of 0.004 in favor of the radiotherapy arm, thus meeting the alpha spending function of *P* = 0.008. The Data and Safety Monitoring Committee (DSMC) and recommended July 31, 2012 to close the 2 treatment arms (R-CHOP-21 and R-CHOP-14) without radiotherapy and to continue both arms with radiotherapy (R-CHOP-21 with radiotherapy and R-CHOP-14 with radiotherapy) as planned. For more details, we refer to the separate publication of the UNFOLDER trial.^14^

Characteristics of patients were compared by χ2 tests and, if necessary, by Fisher exact tests. Treatment duration and dose reductions were assessed using a Kaplan-Meier like estimator.^15^ Response and relapse rates were presented with 95% confidence intervals (CIs). Dose-densification (14 versus 21 days) and radiotherapy (radiotherapy versus observation) were analyzed for EFS, PFS, and OS using Kaplan-Meier plots and log-rank tests. Multivariable Cox regression models adjusted for strata were performed (LDH, stage, and extralymphatic involvement). Hazard ratios with 95% CI were presented. The significance level was 2-sided at 0.05. Statistical analyses were done with SPSS (version 24/25/26/28). LT, MZ, ML, VP, and GH had full access to all the data in the study and final responsibility for the decision to submit for publication.

## RESULTS

From January 02, 2006 to November 16, 2015, a total of 700 patients were enrolled in the UNFOLDER trial, and 467 patients (median age, 44 y, male: 56%, aaIPI of 1: 79%, 131 with PMBCL) were qualified for radiotherapy defined by initial bulky disease and/or extralymphatic involvement. Five additional PMBCL patients did not qualify for radiotherapy (no bulky disease ≥7.5 cm and no extralymphatic involvement [n = 4], bulk surgically removed and no extralymphatic involvement [n = 1]) and were excluded from this analysis (Suppl. Figure S1). None of these patients had an EFS, PFS, or OS event after 6× R-CHOP-21 (n = 2) or 6× R-CHOP-14 (n = 3).

Here we report on 131 patients diagnosed with PMBCL qualifying for radiotherapy. Totally 82 patients (R-CHOP-21: 43 and R-CHOP-14: 39) were randomly assigned to receive radiotherapy and 49 patients (R-CHOP-21: 27 and R-CHOP-14: 22) were assigned to observation without radiotherapy. The imbalance was due to the decision of the DSMC to close the 2 treatment arms (R-CHOP-21 and R-CHOP-14) without radiotherapy as a consequence of the interim analysis described earlier.

Baseline characteristics were balanced between treatment arms (Table 1). The PMBCL patients were young with a median age of 34 years. They were initially staged aaIPI 0 with bulk or aaIPI 1 by the treating physicians and entered into the trial accordingly. During data cleaning, the staging was later corrected to aaIPI 2 for 2 patients. Seventy-nine percent of the patients had LDH above the ULN and 20% twice above the ULN. More than half of the patients (56%) had bulk localizations with a diameter exceeding 10 cm. However, there was a significant difference in the characteristics of patients with PMBCL compared with the other entities of aggressive lymphoma included in the UNFOLDER trial. Patients with PMBCL were more often female (54% versus 40%; *P* = 0.0061), typically younger (median age 34 versus 49 years; *P* < 0.0001), presented more often with LDH above the upper normal value (79% versus 32%; *P* < 0.0001), bulky disease (99% versus 68%; *P* < 0.0001), B symptoms (35% vs 22%; *P* = 0.0027), and less often with advanced stage III or IV (8% versus 46%; *P* < 0.0001) and extralymphatic involvement (24% versus 63%; *P* < 0.0001). No differences were observed in prognostic presentation according to the aaIPI. Similar results emerged when comparing PMBCL not only to patients qualifying for radiotherapy but also to all patients included in the trial (Suppl. Table S3).

**Table 1:** Baseline Demographic and Disease Characteristics of PMBCL and Non-PMBCL Patients Qualifying for Radiotherapy in the UNFOLDER Trial

	R-CHOP 21 (n = 27)		R-CHOP 14 (n = 22)		R-CHOP 21+RTh (n = 43)		R-CHOP 14+RTh (n = 39)		PMBCL (n = 131)		non-PMBCL (n = 336)		*P*-value^*a*^
Male	14	(52%)	11	(50%)	19	(44%)	16	(41%)	60	(46%)	201	(60%)	0.0061
Female	13	(48%)	11	(50%)	24	(56%)	23	(59%)	71	(54%)	135	(40%)	
Age, median (range)	38	(22–60)	32	(20–51)	33	(19–55)	34	(18–60)	34	(18–60)	49	(18–60)	<0.0001
LDH > upper limit of normal	22	(82%)	20	(91%)	32	(74%)	29	(74%)	103	(79%)	108	(32%)	<0.0001
Eastern Cooperative Oncology Group Performance status >1	0	(0%)	0	(0%)	0	(0%)	0	(0%)	0	(0%)	2	(1%)	1.0000
Stage III/IV	1	(4%)	0	(0%)	5	(12%)	4	(10%)	10	(8%)	155	(46%)	<0.0001
Age-adjusted International Prognostic Index													
0 1 2	4 23 0	(15%) (85%) (0%)	2 20 0	(9%) (91%) (0%)	8 33 2	(19%) (77%) (5%)	6 33 0	(15%) (85%) (0%)	20 109 2	(15%) (83%) (2%)	74 259 3	(22%) (77%) (1%)	0.23
Stage I II III IV	17 9 0 1	(63%) (33%) (0%) (4%)	10 12 0 0	(46%) (54%) (0%) (0%)	19 19 0 5	(44%) (44%) (0%) (12%)	13 22 2 2	(33%) (56%) (5%) (5%)	59 62 2 8	(45%) (47%) (2%) (6%)	63 118 45 110	(19%) (35%) (13%) (33%)	<0.0001
Extralymph. Involv.	4	(15%)	4	(18%)	12	(28%)	11	(28%)	31	(24%)	211	(63%)	<0.0001
Extralymph. Involv. >1	1	(4%)	0	(0%)	3	(7%)	0	(0%)	4	(3%)	84	(25%)	<0.0001
Bulk ≥7.5 cm	27	(100%)	21	(96%)	43	(100%)	39	(100%)	130	(99%)	227	(68%)	<0.0001
B symptoms	11	(41%)	5	(23%)	14	(33%)	16	(41%)	46	(35%)	72	(22%)	0.0027
Bone marrow involvement	0	(0%)	0	(0%)	0	(0%)	0	(0%)	0	(0%)	22	(6%)	0.0027
LDH: Normal Elevated Above twice above upper limit of normal	5 15 7	(18%) (56%) (26%)	2 13 7	(9%) (59%) (32%)	11 27 5	(26%) (63%) (12%)	10 22 7	(26%) (56%) (18%)	28 77 26	(21%) (59%) (20%)	228 94 14	(68%) (28%) (4%)	<0.0001
Bulk size: <7.5 cm ≥7.5 cm >10 cm >15 cm	0 16 10 1	(0%) (59%) (37%) (4%)	1 8 12 1	(4%) (36%) (54%) (4%)	0 18 20 5	(0%) (42%) (46%) (12%)	0 15 21 3	(0%) (38%) (54%) (8%)	1 57 63 10	(1%) (44%) (48%) (8%)	109^*b*^ 138 72 16	(32%) (41%) (22%) (5%)	<0.0001

Bone marrow is counted as extralymphatic; and spleen and waldeyers ring are counted as lymphathic.

^*a*^*P*-value for comparison of all PMBCL vs all non-PMBCL.

^*b*^One missing value.

CHOP = cyclophosphamide, doxorubicin, vincristine, and prednisone; LDH = lactate dehydrogenase; PMBCL = primary mediastinal B-cell lymphoma; R-CHOP = rituximab, cyclophosphamide, doxorubicin, vincristine, and prednisone; RTh = radiotherapy.

Extralymphatic involvements were typically close to the bulky mediastinal mass affecting the lung, pleura, pericard, thyroid gland, and soft tissue (Suppl. Table S4).

**Figure 1.**
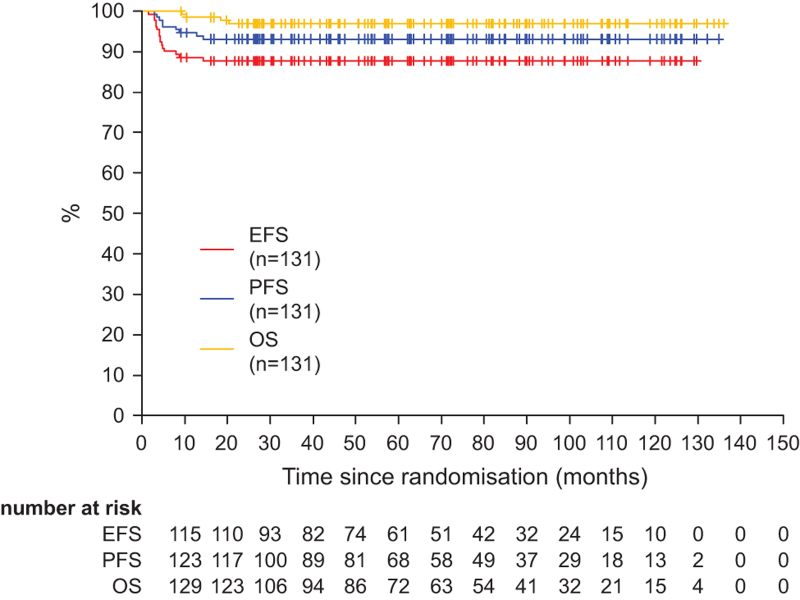
**Event-free, progression-free, and overall survival.** Graph shows EFS, PFS, and OS for all PMBCL patients (n = 131). EFS = event-free survival; OS = overall survival; PFS = progression-free survival; PMBCL = primary mediastinal B-cell lymphoma.

After a median follow-up of 5.5 years, the 3 years event rates were 88% for EFS (95% CI, 82-93), 93% for PFS (95% CI, 89-97), and 97% for OS (95% CI, 94-100) (Figure 1). Importantly, no events were observed later than 20 months with median times of observation exceeding 5 years. In total only 4 patients died, 3 tumor-related and 1 due to concomitant disease (Suppl. Table S5).

**Figure 2.**
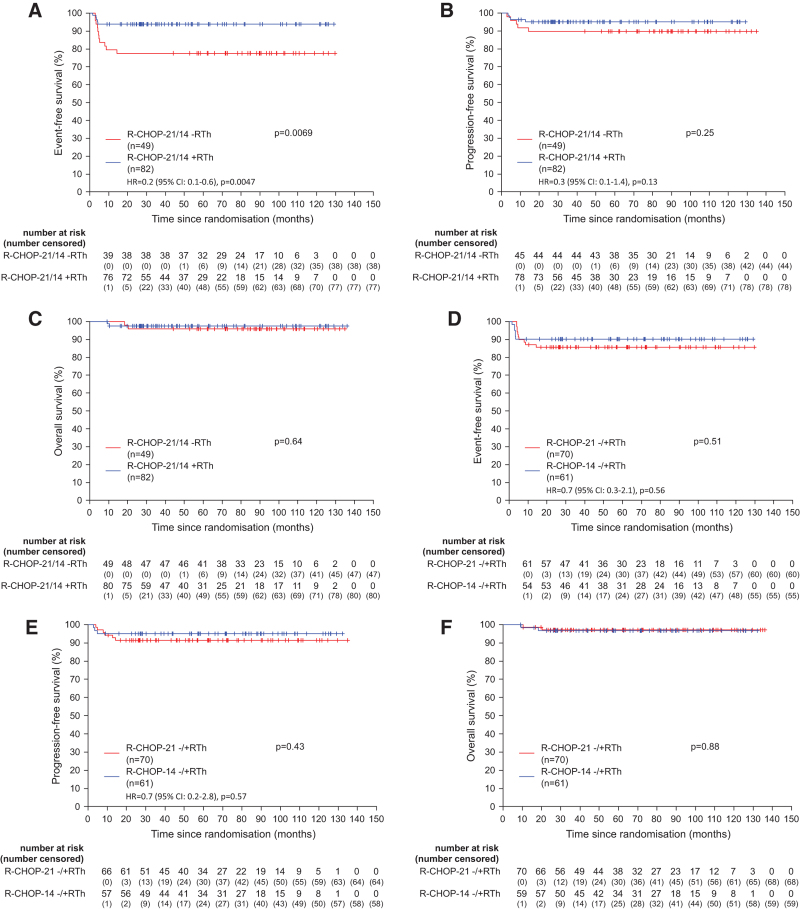
**Event-free, progression-free, and overall survival according to the therapy arm.** Graphs show event-free (A), progression-free (B), and overall survival (C) according to the radiotherapy arm or the observation arm (radiotherapy vs observation) and event-free (D), progression-free (E), and overall survival (F) according to the dose-densification (14 vs 21 d) for all PMBCL patients (n = 131). Hazard ratios for treatment effect adjusted for strata are presented for event-free and PFS. Due to the low number of events, adjusted hazard ratios for OS are not presented. OS = overall survival; PFS = progression-free survival; PMBCL = primary mediastinal B-cell lymphoma; R-CHOP = rituximab, cyclophosphamide, doxorubicin, vincristine, and prednisone; RTh = radiotherapy.

When comparing patients with regard to consolidative radiotherapy or observation, differences in response rates were observed. The rate of CR/CRu was 94% (77/82) versus 84% (41/49), rate of PR was 2% (2/82) versus 10% (5/49) in the radiotherapy arm versus observation arm, respectively (Suppl. Table S6). EFS was superior in patients assigned to receive radiotherapy with 3-year EFS rates being 94% ([95% CI, 89-99] versus 78% [95% CI, 66-89]; *P* = 0.0069) in observation arm (Figure 2A). These differences, however, did not translate into significant different PFS or OS rates. The 3-year PFS of patients assigned to revive radiotherapy versus observation was not significantly different (95% [95% CI, 90-100] versus 90% [95% CI, 81-98]; *P* = 0.25). Also 3-year OS (98% [95% CI, 94-100] versus 96% [95% CI, 90-100]; *P* = 0.64) was not different (Figure 2B and 2C). When comparing patients with regard to dose-densification, EFS, PFS, and OS after R-CHOP-14 and R-CHOP-21 were not different between patients receiving therapy at 2- or 3-week intervals (Figure 2D–2F). These results were also confirmed in multivariable analyses adjusted for the strata. Adherence to dose-densification and dose delivery was strict in all arms with only minor deviations from protocol (Suppl. Figure S2A-S2C). The acute toxicity was very similar in the treatment arms (Suppl. Table S7).

These results were also confirmed in multivariable analyses adjusted for the strata.

Analyzing EFS, PFS, and OS in all 4 arms separately revealed a very similar pattern (Suppl. Figure S3A-S3C). In this exploratory analysis, EFS of the R-CHOP-21 observation arm was significantly inferior compared with both arms with radiotherapy, R-CHOP-21 + radiotherapy and R-CHOP-14 + radiotherapy, respectively (Suppl. Table S8). However, no difference in PFS nor OS was observed between the 4 arms.

Of 118 patients achieving a CR/CRu, 3 patients relapsed, all of them were in the R-CHOP-21 observation arm and one of these patients died after allogeneic stem cell transplantation (SCT) (Table 2 and Suppl. Table S5).

**Table 2:** Events in PMBCL Patients

No.	Therapy Arm	Response at the End of Treatment	Further Therapy	EFS Event	PFS Event	Further Therapy	Survival Status; Survival Time (mo)
1	R-CHOP-21	Partial response	Radiotherapy	Partial response at the end of study therapy	No		Alive (135 mo)
2	R-CHOP-21	Partial response	Radiotherapy	Partial response at the end of study therapy	No		Alive (109 mo)
3	R-CHOP-21	CR/CRu +additional treatment	Radiotherapy	Application of additional treatment	No		Alive (120 mo)
4	R-CHOP-21	Progressive disease		progressive disease	Progressive disease	Autologous stem cell transplantation	Alive (113 mo)
5	R-CHOP-21	CR/CRu		Relapse	Relapse	Radiotherapy	Alive (94 mo)
6	R-CHOP-21	CR/CRu		Relapse	Relapse	Autologous stem cell transplantation +radiotherapy	Alive (87 mo)
7	R-CHOP-21	CR/CRu		Relapse	Relapse	Allogenic stem cell transplantation	Death (20 mo)
8	R-CHOP-21	Partial response	Radiotherapy	Partial response at the end of study therapy	No		Alive (81 mo)
9	R-CHOP-14	Partial response	Autologous stem cell transplantation +radiotherapy	Partial response at the end of study therapy	No		Alive (132 mo)
10	R-CHOP-14	Progressive disease		Progressive disease	Progressive disease	Burkitt protocol	Death (18 mo)
11	R-CHOP-14	Partial response	Radiotherapy	Partial response at the end of study therapy	No		Alive (88 mo)
12	R-CHOP-21 + radiotherapy	Partial response	2x R-CHOP+radiotherapy	Partial response at the end of study therapy	Relapse	Autologous stem cell transplantation	Alive (134 mo)
13	R-CHOP-21 + radiotherapy	Progressive disease		Progressive disease	Progressive disease	Autologous stem cell transplantation	Death (10 mo)
14	R-CHOP-14 + radiotherapy	Unknown	Auto SCT + rituximab consolidation	Unknown response at the end of study therapy, application of additional treatment	No		Alive (107 mo)
15	R-CHOP-14 + radiotherapy	Progressive disease		Progressive disease	Progressive disease	Autologous stem cell transplantation +radiotherapy, allogenic stem cell transplantation, lymphocytes	Alive (51 mo)
16	R-CHOP-14 + radiotherapy	Partial response		Partial response at the end of study therapy	Progressive disease	Unknown	Death (9 mo)

CR/CRu = complete response/unconfirmed complete response; EFS =event-free survival; PFS = progression-free survival; PMBCL = primary mediastinal B-cell lymphoma; R-CHOP = rituximab, cyclophosphamide, doxorubicin, vincristine, and prednisone; SCT = stem cell transplantation.

Protocol adherence in the radiotherapy arms was high. Patients randomized to the observation arm did not actually receive radiotherapy except for last 2 patients recruited, who received radiotherapy after the decision of the DSMC to close the 2 treatment arms (R-CHOP-21 and R-CHOP-14) without radiotherapy as a consequence of the interim analysis described above; 76 of 82 (93%) patients randomized in the radiotherapy arms received radiotherapy according to the protocol. Six patients did not receive radiotherapy as planned, 1 due to patient’s wish, 3 due to insufficient response, 1 due to excessive toxicity, and 1 due to protocol violation, respectively (Table 3).

**Table 3:** Protocol Adherence of Radiotherapy in PMBCL Patients

	R-CHOP-21 (n = 27)		R-CHOP-14 (n = 22)		R-CHOP-21 + Radiotherapy (n = 43)		R-CHOP-14 + Radiotherapy (n=39)	
Radiotherapy yes								
According to protocol	-		-		39	(91%)	37	(95%)
Due to interim analysis^*a*^	1	(4%)	1	(4%)	-		-	
Radiotherapy no								
According to the protocol	26	(96%)	21	(95%)	-		-	
Insufficient response	-		-		2	(5%)	1	(3%)
Excessive toxicity	-		-		1	(2%)	-	
Protocol violation	-		-		-		1	(3%)^*b*^
Patients decision	-		-		1	(2%)	-	

^*a*^After interim analysis, the observation arms were closed and radiotherapy was performed also in the observation arms.

^*b*^Change of treatment to autologous stem cell transplantation after 4 cycles of R-CHOP.

PMBCL = primary mediastinal B-cell lymphoma; R-CHOP = rituximab, cyclophosphamide, doxorubicin, vincristine, and prednisone.

However, physicians observed an CRu or a PR at the end of the planned treatment in 5 patients treated within the observation arms and subsequently decided to administer radiotherapy to these patients. Applied radiotherapy was subsequently counted as an EFS-event. These patients were then restaged as CR after completion of radiotherapy (Table 2).

Seven patients in the observation arms received consolidative radiotherapy. Within the R-CHOP-21 arm, 3 patients received radiotherapy after achieving a partial remission, 1 patient after a CR/CRu, and 1 patient after the decision of the DSMC to close the 2 treatment arms (R-CHOP-21 and R-CHOP-14) without radiotherapy as a consequence of the interim analysis described earlier. Within the R-CHOP-14 arm, 1 patient each received radiotherapy after achieving a PR and 1 patient after the decision of the DSMC to close the 2 treatment arms (R-CHOP-21 and R-CHOP-14) without radiotherapy as a consequence of the interim analysis described above. In a post hoc as-treated analysis, these patients were analyzed within the radiotherapy arm. The 3-year PFS with radiotherapy was slightly but not significantly better in comparison to observation without radiotherapy (96% [95% CI, 91-100] versus 88% [95% CI, 78-98]; *P* = 0.12; Suppl. Figure S4A). OS was not different in the as-treated analysis (*P* = 0.47) (Suppl. Figure S4B).

In 76 patients who received radiotherapy as randomized toxicity was moderate (Suppl. Table S9). Oesophagitis/dysphagia common toxicity criteria (CTC) grade 3 or 4 in 6% was the only relevant observed local toxicity. Hematological toxicities grade 3 or 4 were seen in 7% patients for leukocytopenia and for 1 patient in thrombocytopenia. Cardiac long-term toxicities (eg, coronary heart diseases) have not been observed.

Among the 131 patients, only 2 developed a secondary neoplasia so far. Both patients were in the R-CHOP-21 arm with additional radiotherapy. One was a 45-year-old patient with prostate cancer occurring 3 years after randomization and 1 a 49-year-old male patient with tonsillar carcinoma diagnosed 1 year after randomization.

Among the 20 patients with aaIPI 0, no EFS, PFS, or OS event was observed, that is, all these values were 100% for over 5 years. All EFS (n = 16), PFS (n = 9), or OS events (n = 4) observed occurred among patients with aaIPI 1 (n = 109) or aaIPI 2 (n = 2) within a period of 20 months after randomization.

**Figure 3.**
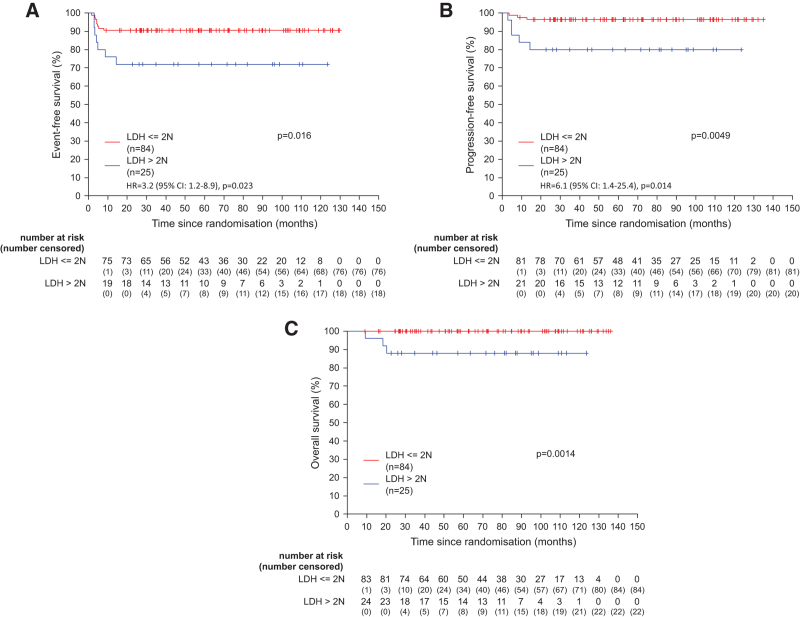
**Event-free, progression-free, and overall survival according to the LDH groups.** Graphs show event-free (A), progression-free (B), and overall survival (C) according to the LDH groups (above twice above the ULN yes/no) for all PMBCL patients with aaIPI = 1 (n = 109). Univariate hazard ratios are presented for event-free survival and PFS. Due to the low number of events, hazard ratio for OS is not presented. LDH = lactate dehydrogenase; OS = overall survival; PFS = progression-free survival; PMBCL = primary mediastinal B-cell lymphoma; ULN = upper limit of normal.

LDH is one of the prognostic factors considered in the aaIPI score. At staging, LDH was elevated above ULN in 79% of the patients (Table 1). This was the major contributor to the classifications of patients to aaIPI 1 (101 from 109 patient [93%]), whereas only 8 [7%] were classified as aaIPI 1 due to the presentation with Ann Arbor stage III/IV. In patients with aaIPI 1 and LDH values twice above the ULN was associated with a significant inferior EFS, PFS, and OS (Figure 3A–3C).

## DISCUSSION

The PMBCL cohort analyzed here is unique by its number and by its nature as a prospective and randomized subgroup within a clinical trial. The patients with PMBCL treated within the UNFOLDER trial had an excellent prognosis with 6× R-CHOP. Of 131 randomized for R-CHOP-14 versus 21 and for radiotherapy versus omission of radiotherapy, only 4 patients died (5.5 y median time of observation) resulting in an OS of 97% after 3 years. The PFS was 93% after 3 years with no relapses observed thereafter. This observed absence of late relapses is consistent with previous studies.^4^ The excellent outcome is remarkable. First, the trial was a multicenter study recruiting in 4 different countries, which minimizes the risk for positively selecting patients with a better prognosis, possibly inherent in single-center studies. Second, 79% of patients presented with elevated LDH, an adverse prognostic factor of the International Prognostic Index, which reliably separates patients into distinct prognostic subgroups.^16,17^ The results imply a more pronounced sensitivity to immuno-chemotherapy of PMBCLs compared with other aggressive B-cell Non-Hodgkin lymphoma, probably due to the particularly different lymphoma biology.^2,18–22^

As seen in previous trials, an elevated LDH is a frequent finding in PMBCL, but plays apparently a minor prognostic role here.^23^ However, LDH increased twice above the ULN has been proposed in previous studies as a more meaningful negative prognostic marker for PMBCL.^24^

Our results reveal no differences in outcome and toxicity between 6× R-CHOP-14 and 6× R-CHOP-21 among PMBCL. We could not confirm an exploratory subgroup analysis of the UK NCRI R-CHOP-21, which demonstrated a nonsignificant trend to better PFS and OS in 50 patients with PMBCL treated with R-CHOP-14 prospectively.^25^ Neither do our results support a retrospective analysis of the Lymphoma Study Association (LYSA), which demonstrated inferior PFS and OS of R-CHOP-21 compared with R-CHOP-14 or ACVBP.^26^ Rather, the results are in concordance with the overall outcome on the chemotherapy comparison in the UNFOLDER trial. Hence, we conclude that the choice of 6× R-CHOP-14 or 6× R-CHOP-21 is equally justified for PMBCL. However, this conclusion is somehow limited by the exclusion of patients with aaIPI >1. In addition, our results might advocate a different approach for patients with LDH increased twice above the ULN. Whether DA-EPOCH-R, which provided excellent results in phase-II study of 51 patients, is superior compared with R-CHOP is difficult to answer in the absence of a randomized trial.

The UNFOLDER trial, well-balanced for baseline characteristics, shows the clear advantages of its randomized and prospective nature. In a retrospective study of the LYSA described outcome after the first-line treatment. In the R-ACVBP and R-CHOP-14 cohorts, 27.3% and 38.2% were treated with consolidative ASCT compared with 1.8% in the R-CHOP-21 cohort, and there were also more consolidating radiotherapies in patients treated with R-CHOP-14 (21.1%) compared with the R-CHOP-21 (3.5%).^26^ These represent clear biases of retrospective analyses, which are not present in the UNFOLDER PMBCL subgroup analysis.

Regarding consolidative radiotherapy patients assigned to observation only had a significant inferior EFS. Based on the CT-scans, physicians classified their response after the end of the planned treatment more frequently as a PR than in the treatment arm with radiotherapy. The majority of patients judged as PR after chemotherapy were then treated with additional radiotherapy. Likewise, there were relapses after CRu, which were treated with radiotherapy. Due to these response-adapted radiotherapy, 4 of 4 PRs in the observation arm and 1 of 3 relapses were converted into CR (Table 2). As a result of this practice, the PFS and OS was not significantly different in both treatment arms.

The very favorable outcome of PMBCL to R-CHOP treatment or similar regimens suggests that radiotherapy seems not to be required to all patients.^6^ Indeed, the majority of patients allocated to the observation arm were in a continuous remission without any radiotherapy as indicated by an EFS of 78% after 3 years. Thus, radiotherapy might be spared to patients achieving a CR after a R-CHOP-based regimen. This hypothesis is investigated currently in the ongoing International Extranodal Lymphoma Study Group 37 (IELSG-37) trial, assessing the role of radiotherapy in patients achieving a PET-negative CR (ClinicalTrials.gov Identifier: NCT01599559). However, our results suggest a benefit of radiotherapy for those patients who are responding poorly.

As progressive disease and relapses occur early, radiotherapy should already be applied to poor responders as part of the first-line therapy. However, this decision could possibly be limited to FDG-PET-positive residuals. A registry study from British Columbia comes to a similar conclusion. FDG-PET after R-CHOP was used to guide radiotherapy and only those with a PET-positive scan defined as Deauville score 4–5 received radiotherapy. Before the availability of FDG-PET in 2005, all patients with PMBCL were recommended to receive radiotherapy. Time to progression and OS were similar across the 2 treatment eras. However, the PET-adapted approach reduced radiotherapy in the majority with only 28% of patients receiving radiotherapy.^27^ Investigating radiotherapy in PET-positive residual tumors in PMBCL in a randomized trial is warranted, ideally applying radiotherapy in a short interval after the last cycle of chemotherapy.

Our results have several limitations. First, as stated earlier at the time the trial was designed and conducted, we had no access to routine FDG/PET-CT-scans and trial imaging was largely based on the CT-scans. FDG/PET examination in patients with CRu and PR after 6× R-CHOP would be a justified procedure to investigate the need of an additional consolidating radiotherapy.

Second, the trial arms are not equally large due to the decision to stop randomization for radiotherapy by the DSMC. Hence, the arms without radiotherapy were abandoned and radiotherapy was given to all bulky and extranodal sites for the remaining participants. This decision was based on the analysis of EFS as the primary end point. In this light, PFS has been proposed as the preferred end point in lymphoma clinical trials.^28^

Third, exclusion of patients with aaIPI >1 may have partially contributed to the excellent reported outcome.

Finally, in the meantime new potential therapeutic options for r/r PMBCL emerged, improving outcome of the relatively rare refractory or relapsed cases.^7–9,11^

However, the strength of this study is its phase-3 design. Randomized trials are providing the highest degree of scientific evidence about efficacy of a certain therapeutic strategy and are very scarce in defining the role of radiotherapy in aggressive lymphoma in the era of rituximab.

Despite the limitation in a pre-PET-era trial, in summary overall outcomes of PMBCL patients treated with R-CHOP are favorable with a 3 and 5 year OS of 97%. Radiotherapy does not seem to be required as routine treatment of these patients unless in case of PR or relapse, with results of prospective PET-driven trials being awaited. Elevated LDH is a common feature in PMBCL but only prognostic, when elevated twice above the ULN. This finding might be an important baseline characteristic for identifying high-risk PMBCL for future trials.

## ACKNOWLEDGMENTS

We thank the patients, families, caregivers, and principal investigators of all countries who participated in the clinical trial including Peter de Nully Brown (Denmark); Massimo Federico (Italy); Ofer Shpilberg (Israel); and Michael Pfreundschuh (Germany; died in March 2018), who designed the study, wrote the protocol, was the principal investigator in Germany, and was chairman of the study. We thank the numerous research and trial groups, including the German High-Grade Non-Hodgkin’s Lymphoma Study Group, the Fondazione Italiana Linfomi, and the Nordic lymphoma group for their participation in the trial. We thank the international board of expert pathologists who provided histopathological review: Andreas Rosenwald (chairman; Wuerzburg, Germany); Alfred C. Feller (Luebeck, Germany); Martin-Leo Hansmann (Frankfurt, Germany); Wolfram Klapper (Kiel, Germany); Peter Moeller (Ulm, Germany); Hans Konrad Mueller-Hermelink (Wuerzburg, Germany); Elisabeth Ralfkiaer (Copenhagen, Denmark); Harald Stein (Berlin, Germany); Philippe Trougouboff (Afula, Israel); and Hans-Heinrich Wacker (Kiel, Germany). We thank Christian Berdel, Jochen Fleckenstein, and Christian Ruebe for imaging review as independent expert for radiology and radiation oncology at Homburg/Saar, Germany. We thank the data and safety monitoring committee that served as an independent expert advisory group to assess safety and efficacy data during the trial, including Günter Brittinger (Essen, Germany, died June, 2021); Volker Diehl (Koeln, Germany); and Klaus Havemann (Marburg, Germany, died May, 2016). We thank the study trial office Homburg/Saar, Germany, including the secretaries Waltraud Beck (died August, 2013) and Daniela Ehlert; the data management team Stephanie Angel, Elina Dick, Kerstin Höhn, Kirstin Monz, Tanja Rixecker, and Christian Schorpp; and the clinicians Josif Amam, Konstantinos Christofyllakis, Gerhard Held, Niels Murawski, Milena Pfeiffer, Viola Poeschel, Christoph Renner, Rudolf Schmits (died October, 2016), Jörg Schubert, Pia Sweet, Anne Wolf, and Carsten Zwick. We thank the data center in Leipzig, Germany, including the database team Sigrid Haupt, Jürgen Hentschel, Martina Kunert, Beate Mann, Katja Rillich, Ulrike Schoenwiese, and Barbara Wicklein; and the biometry team Bettina Altmann, Markus Loeffler, and Marita Ziepert.

## AUTHOR CONTRIBUTIONS

GH and VP oversaw the study (originally designed by Michael Pfreundschuh) and contributed to study design, data monitoring, data interpretation, and writing and approval of the report. L. Thurner contributed to data interpretation, and writing and approval of the report. ML and MZ did the statistical analysis and contributed to study design, data interpretation, and writing and approval of the report. GO contributed to data interpretation and reference pathology. AR coordinated the reference pathology. CB and JF coordinated and assessed the imaging and radiation oncology review and contributed to data interpretation. HS contributed to study oversight and data interpretation. OS was the principal investigator in Israel and recruited patients. MF was the principal investigator in Italy and recruited patients. PdNB was the principal investigator in Denmark and recruited patients. ME contributed to study oversight. NM contributed to study oversight and data monitoring. SS contributed to data interpretation and recruited patients. BG, MN, NS, GW, and L. Truemper contributed to study design and recruited patients. BA contributed to statistical analysis and data interpretation. GH, L. Thurner, CS, KC, AV, PB, WE-R, NF, MW-H, FG, BK-S, AN, and MB recruited patients. All authors have reviewed and approved the final version of the report.

## DATA SHARING STATEMENT

Data of results reported in this article will be shared after de-identification. Individual, pseudonymised data and data dictionaries will be available upon request up to 5 years after publication after providing a data sharing agreement directed to dshnhl@uks.eu, which describes intended analyses and required data. Selected data will be also available on the Leipzig Health Atlas (https://www.health-atlas.de).

## DISCLOSURES

GH has received grants from Roche and Bristol-Myers Squibb and personal fees from Bristol-Myers Squibb, Roche, Amgen, Spectrum and MSD. L Thurner has received travel grants from Abbvie, Janssen and EUSA-Pharm, and has indicated consultancy for Takeda, Astra-Zeneca, Merck, EUSA-pharm. VP has received grants from Deutsche Krebshilfe (German Cancer Aid), Chugai, Abbvie, Amgen, Roche, and Bristol-Myers Squibb. AV has received honoraria from Roche, Amgen, Kite, Gilead, Novartis, Bristol-Myers Squibb and has indicated a membership of the advisory board of Roche, Amgen, Kite, Gilead, Novartis, Bristol-Myers Squibb. MN has received travel grants from Roche, Celgene, and MSD and personal fees from Roche, Celgene, MSD, Janssen, Amgen, Incyte, and Abbvie. FG participates in advisory board of Roche, Boehringer Ingelheim, Abbvie, Merck, Takeda, MSD, Sanofi, Pfizer, Novartis, Amgen, and Janssen. PdNB has indicated consultancy for Roche, Incyte, and Novartis. SS has received grants from Abbvie, Astra-Zeneca, Celgene, Gilead, Roche, Janssen, Novartis, Morphosys and has indicated consultancy for for Abbvie, Astra-Zeneca, Celgene, Gilead, Roche, Janssen, Novartis, Morphosys; he has received drug/equipment supplied by entity from Abbvie, Astra-Zeneca, Celgene, Gilead, Roche, Janssen, Novartis, Morphosys. All the other authors have no conflicts of interest to disclose.

## SOURCES OF FUNDING

The trial was supported by a grant from the German nonprofit foundation, Deutsche Krebshilfe (German Cancer Aid), reference number 106377 and Chugai Pharmaceuticals.

## Supplementary Material



## References

[1] Swerdlow SH Campo E Harris NL. WHO Classification of Tumours of Haematopoietic and Lymphoid Tissues. Vol. 2. 4th ed. International Agency for Research on Cancer (IARC); 2017.

[2] Bea S Zettl A Wright G. Diffuse large B-cell lymphoma subgroups have distinct genetic profiles that influence tumor biology and improve gene-expression-based survival prediction. Blood. 2005;106:3183–3190. 16046532 10.1182/blood-2005-04-1399 PMC1895326

[3] Zinzani PL Martelli M Bertini M. Induction chemotherapy strategies for primary mediastinal large B-cell lymphoma with sclerosis: a retrospective multinational study on 426 previously untreated patients. Haematologica. 2002;87:1258–1264. 12495899

[4] Rieger M Österborg A Pettengell R. Primary mediastinal B-cell lymphoma treated with CHOP-like chemotherapy with or without rituximab: results of the Mabthera International Trial Group study. Ann Oncol. 2011;22:664–670. 20724576 10.1093/annonc/mdq418

[5] Gleeson M Hawkes EA Cunningham D. Rituximab, cyclophosphamide, doxorubicin, vincristine and prednisolone (R-CHOP) in the management of primary mediastinal B-cell lymphoma: a subgroup analysis of the UK NCRI R-CHOP 14 versus 21 trial. Br J Haematol. 2016;175:668–672. 27477167 10.1111/bjh.14287

[6] Dunleavy K Pittaluga S Maeda LS. Dose-adjusted EPOCH-rituximab therapy in primary mediastinal B-cell lymphoma. N Engl J Med. 2013;368:1408–1416. 23574119 10.1056/NEJMoa1214561 PMC4568999

[7] Locke FL Miklos DB Jacobson CA. Axicabtagene ciloleucel as second-line therapy for large B-cell lymphoma. N Engl J Med. 2022;386:640–654. 34891224 10.1056/NEJMoa2116133

[8] Kamdar M Solomon SR Arnason J. Lisocabtagene maraleucel versus standard of care with salvage chemotherapy followed by autologous stem cell transplantation as second-line treatment in patients with relapsed or refractory large B-cell lymphoma (TRANSFORM): results from an interim analysis of an open-label, randomised, phase 3 trial. Lancet. 2022;399:2294–2308. 35717989 10.1016/S0140-6736(22)00662-6

[9] Bishop MR Dickinson M Purtill D. Second-line tisagenlecleucel or standard care in aggressive B-cell lymphoma. N Engl J Med. 2022;386:629–639. 34904798 10.1056/NEJMoa2116596

[10] Dickinson MJ Carlo-Stella C Morschhauser F. Glofitamab for relapsed or refractory diffuse large B-cell lymphoma. N Engl J Med. 2022;387:2220–2231. 36507690 10.1056/NEJMoa2206913

[11] Zinzani PL Santoro A Gritti G. Nivolumab combined with brentuximab vedotin for relapsed/refractory primary mediastinal large B-cell lymphoma: efficacy and safety from the phase II checkmate 436 study. J Clin Oncol. 2019;37:3081–3089. 31398081 10.1200/JCO.19.01492 PMC6864847

[12] Thieblemont C Phillips T Ghesquieres H. Epcoritamab, a novel, subcutaneous CD3xCD20 bispecific T-cell-engaging antibody, in relapsed or refractory large B-cell lymphoma: dose expansion in a phase I/II trial. J Clin Oncol. 2023;41:2238–2247. 36548927 10.1200/JCO.22.01725 PMC10115554

[13] Cheson BD Horning SJ Coiffier B. Report of an international workshop to standardize response criteria for non-Hodgkin’s lymphomas. J Clin Oncol. 1999;17:1244–1244. 10561185 10.1200/JCO.1999.17.4.1244

[14] Thurner L Ziepert M Berdel C. Radiation and dose-densification of R-CHOP in aggressive B-cell lymphoma with intermediate prognosis: the UNFOLDER study. 2022. 10.1097/HS9.0000000000000904 PMC10325769 37427146

[15] Wunderlich A Kloess M Reiser M. Practicability and acute haematological toxicity of 2- and 3-weekly CHOP and CHOEP chemotherapy for aggressive non-Hodgkin’s lymphoma: results from the NHL-B trial of the German High-Grade Non-Hodgkin’s Lymphoma Study Group (DSHNHL). Ann Oncol. 2003;14:881–893. 12796026 10.1093/annonc/mdg249

[16] International Non-Hodgkin's Lymphoma Prognostic Factors Project. A predictive model for aggressive non-Hodgkin’s lymphoma. N Engl J Med. 1993;329:987–994. 8141877 10.1056/NEJM199309303291402

[17] Ziepert M Hasenclever D Kuhnt E. Standard international prognostic index remains a valid predictor of outcome for patients with aggressive CD20+ B-Cell lymphoma in the rituximab era. J Clin Oncol. 2010;28:2373–2380. 20385988 10.1200/JCO.2009.26.2493

[18] Steidl C Gascoyne RD. The molecular pathogenesis of primary mediastinal large B-cell lymphoma. Blood. 2011;118:2659–2669. 21700770 10.1182/blood-2011-05-326538

[19] Rosenwald A Wright G Leroy K. Molecular diagnosis of primary mediastinal B cell lymphoma identifies a clinically favorable subgroup of diffuse large B cell lymphoma related to Hodgkin lymphoma. J Exp Med. 2003;198:851–862. 12975453 10.1084/jem.20031074 PMC2194208

[20] Savage KJ Monti S Kutok JL. The molecular signature of mediastinal large B-cell lymphoma differs from that of other diffuse large B-cell lymphomas and shares features with classical Hodgkin lymphoma. Blood. 2003;102:3871–3879. 12933571 10.1182/blood-2003-06-1841

[21] Green MR Monti S Rodig SJ. Integrative analysis reveals selective 9p24.1 amplification, increased PD-1 ligand expression, and further induction via JAK2 in nodular sclerosing Hodgkin lymphoma and primary mediastinal large B-cell lymphoma. Blood. 2010;116:3268–3277. 20628145 10.1182/blood-2010-05-282780 PMC2995356

[22] Mottok A Hung SS Chavez EA. Integrative genomic analysis identifies key pathogenic mechanisms in primary mediastinal large B-cell lymphoma. Blood. 2019;134:802–813. 31292115 10.1182/blood.2019001126

[23] Savage KJ Al-Rajhi N Voss N. Favorable outcome of primary mediastinal large B-cell lymphoma in a single institution: the British Columbia experience. Ann Oncol. 2006;17:123–130. 16236753 10.1093/annonc/mdj030

[24] Vassilakopoulos TP Michail M Papageorgiou S. Identification of very low-risk subgroups of patients with primary mediastinal large B-cell lymphoma treated with R-CHOP. Oncologist. 2021;26:597–609. 33870594 10.1002/onco.13789 PMC8265336

[25] Gleeson M Hawkes EA Cunningham D. Rituximab, cyclophosphamide, doxorubicin, vincristine and prednisolone (R-CHOP) in the management of primary mediastinal B-cell lymphoma: a subgroup analysis of the UK NCRI R-CHOP 14 versus 21 trial. Br J Haematol. 2016;175:668–672. 27477167 10.1111/bjh.14287

[26] Camus V Rossi C Sesques P. Outcomes after first-line immunochemotherapy for primary mediastinal B-cell lymphoma: a LYSA study. Blood Adv. 2021;5:3862–3872. 34461634 10.1182/bloodadvances.2021004778 PMC8679665

[27] Hayden AR Tonseth P Lee DG. Outcome of primary mediastinal large B-cell lymphoma using R-CHOP: impact of a PET-adapted approach. Blood. 2020;136:2803–2811. 32603413 10.1182/blood.2019004296

[28] Cheson BD Pfistner B Juweid ME. Revised response criteria for malignant lymphoma. J Clin Oncol. 2007;25:579–586. 17242396 10.1200/JCO.2006.09.2403

