# Supplementary material for: Radiation and Dose-densification of R-CHOP in Primary Mediastinal B-cell Lymphoma: Subgroup Analysis of the UNFOLDER Trial
Source: Hemasphere. 2023 Jul 5;7(7):e917. doi: 10.1097/HS9.0000000000000917 (PMC10325764; doi:10.1097/HS9.0000000000000917)
Supplement: Supplementary file 1 [file hs9-7-e917-s001.docx]

**Supplementary Appendix**

**Radiation and dose-densification of R-CHOP in primary mediastinal B-cell lymphoma: Subgroup analysis of the UNFOLDER trial**

| **Table of content** |  |  |
| --- | --- | --- |
|  |  |  |
| **Supplementary Tables** |  | **Page 3-15** |
|  |  |  |
| **Supplementary Table S1** | List of UNFOLDER study investigators | **Page 3-9** |
| **Supplementary Table S2** | UNFOLDER Protocol Amendments | **Page 10** |
| **Supplementary Table S3** | Baseline patient demographic and disease characteristics of PMBCL and all Non-PMBCL patients in the UNFOLDER trial | **Page 11** |
| **Supplementary Table S4** | Sites of extralymphatic involvement in PMBCL patients | **Page 12** |
| **Supplementary Table S5** | Causes of death in PMBCL | **Page 13** |
| **Supplementary Table S6** | Rates of complete response, progressive disease, and relapse rates in PMBCL | **Page 13** |
| **Supplementary Table S7** | Toxicity of chemoimmunotherapy per patient in PMBCL | **Page 13-14** |
| **Supplementary Table S8** | 3-year event-free, progression-free, and overall survival in PMBCL | **Page 14** |
| **Supplementary Table S9** | Acute Toxicity of radiotherapy in PMBCL | **Page 15** |
|  |  |  |
| **Supplementary Figures** |  |  |
| **Supplementary Figure S1**  **Supplementary Figure S2** | CONSORT diagram of PMBCL subgroup in UNFOLDER  Total duration of CHOP chemotherapy, absolute dose of doxorubicin, and rituximab according to treatment arm for all PMBCL patients | **Page 15**  **Page 16** |
| **Supplementary Figure S3** | Event-free, progression-free and, overall survival according to treatment arm for all PMBCL patients | **Page 17** |
| **Supplementary Figure S4** | Progression-free and, overall survival according to treatment given in radiotherapy- or observation-arm for all PMBCL patients | **Page 18** |
|  |  |  |
| **Study protocol** |  |  |
| **UNFOLDER study protocol** |  | **Page 19-seq.** |

Table S1**:** List of UNFOLDER study investigators

| Principal Investigator | Site location |
| --- | --- |
| Wolfgang Abenhardt | MOPS Elisenhof, Prielmayerstr. 1, 80335 München, GERMANY |
| Reinhard Andreesen | Klinikum der Universität Regensburg, Abteilung Hämatologie und Internistische Onkologie, Franz-Josef-Strauss-Allee 11, 93042 Regensburg, GERMANY |
| Wolfgang Bair | Schloßbergklinik Oberstaufen, Schlossstr. 27, 87534 Oberstaufen/Allgäu, GERMANY |
| Hans Becker | Hans-Susemihl-Krankenhaus Emden, Med. Klinik I, Bolardusstr. 20, 26721 Emden, GERMANY |
| Dirk Behringer | Augusta-Kranken-Anstalt gGmbH, Klinik f. Hämatologie und Onkologie, Bergstr. 26, 44791 Bochum, GERMANY |
| Martin Bentz | Städtisches Klinikum Karlsruhe, II. Med. Klinik, Moltkestr. 90, 76133 Karlsruhe, GERMANY |
| Wolfgang E. Berdel | Universitätsklinikum Münster, Med. Klinik und Poliklinik A, Hämatologie/Onkologie, Albert-Schweitzer-Str. 33, 48129 Münster, GERMANY |
| Lothar Bergmann | Klinikum d. Johann-Wolfgang-Goethe-Univ. Frankfurt, Med. Klinik III, Theodor-Stern-Kai 7, 60590 Frankfurt/Main, GERMANY |
| Harald Biersack | Universitätsklinikum Schleswig-Holstein (Campus Lübeck), Ratzeburger Allee 160, 23538 Lübeck, GERMANY |
| Stephan Bildat | Klinikum Kreis Herford, Med. Versorgungszentrum, Schwarzenmoorstr. 70, 32049 Herford, GERMANY |
| Hans Peter Böck | Gemeinschaftspraxis, Hämatologie u. Intern. Onkologie, Marktstr. 11, 63065 Offenbach, GERMANY |
| Peter Borchmann | Universitätsklinik Köln, Klinik I für Innere Medizin, Kerpener Str. 62, 50937 Köln, GERMANY |
| Jan Braess | Krankenhaus der Barmherzigen Brüder Regensburg, Prüfeninger Str. 86, 93049 Regensburg, GERMANY |
| Stefan Brettner | Kreiskrankenhaus Waldbröl, Dr.-Goldenbogen-Str. 10, 51545 Waldbröl, GERMANY |
| Maura Brugiatelli | Az. Ospedaliera Messina, Contrada Sperone, 98158 Messina, ITALY |
| Friedrich Burghardt | Evang. Krankenhaus Holzminden, Forster Weg 34, 37603 Holzminden |
| Martin Burk | Klinikum Stadt Hanau, Onkologie/Hämatologie, Leimenstr. 20, 63450 Hanau, GERMANY |
| Angelo Michele Carella | AOU San Martino Genova, Largo Rosanna Benzi 10, 16132 Genova, ITALY |
| Beate Dargel | Harz-Klinikum Wernigerode-Blankenburg GmbH, Med. Klinik, Ilsenburger str. 15, 38855 Wernigerode, GERMANY |
| Peter de Nully Brown | Rigshospitalet, Department of Hematology, Blegdamsvej 9, 2100 Kobenhavn, DENMARK |
| Matthias Demandt | MVZ Onkologie, Klinikum Straubing GmbH, St.-Elisabeth-Str. 23, 94315 Straubing, GERMANY |
| Yves Dencausse | Praxis f. Innere Medizin am KH Siloah, Wilferdinger Str. 67, 75179 Pforzheim, GERMANY |
| Judith Dierlamm | Universitätsklinikum Eppendorf, II. Med. Klinik und Poliklinik, Martinistr. 52, 20246 Hamburg, GERMANY |
| Josef Diers | St. Marienhospital Vechta, Innere Medizin, Hämatologie u. Intern. Onkologie, Marienstr. 6-8, 49377 Vechta, GERMANY |
| Hermann Dietzfelbinger | Privatklinik Dr. med. R. Schindlbeck, Seestr. 43, 82211 Herrsching, GERMANY |
| Ulrich Dührsen | Universitätsklinikum Essen, Klinik für Hämatologie, Hufelandstr. 55, 45122 Essen, GERMANY |
| Erik Engel | Hämatologisch Onkologische Praxis Altona, Mörkenstr. 43-47, 22767 Hamburg, GERMANY |
| Walburga Engel-Riedel | Lungenklinik Köln-Merheim, Ostmerheimer Str. 200, 51109 Köln, GERMANY |
| Henning Eschenburg | Gemeinschaftspraxis Dr. S. Duda/ Dr. Eschenburg/ Dr. S. Wilhelm, Am Wall 1, 18273 Güstrow, GERMANY |
| Massimo Federico | CHIMOMO Department, University of Modena and Reggio Emilia, 41100 Modena, ITALY |
| Thomas Fischer | Universitätsklinikum Magdeburg, Zentrum f. Innere Medizin, Klinik f. Hämatologie/Onkologie, Leipziger Str. 44, 39120 Magdeburg, GERMANY |
| Ludwig Fischer von Weikersthal | Klinikum St. Marien Amberg - MVZ, Mariahilfbergweg 7, 92224 Amberg, GERMANY |
| Helmut Forstbauer | Praxis Dr. med. Helmut Forstbauer, Schloßstr. 18, 53840 Troisdorf, GERMANY |
| Norbert Frickhofen | Dr.-Horst-Schmidt-Kliniken Wiesbaden, Innere Medizin III, 65199 Wiesbaden, GERMANY |
| Arnold Ganser | Medizinische Hochschule Hannover, Zentrum f. Innere Medizin, Carl-Neuberg-Str. 1, 30625 Hannover, GERMANY |
| Tobias Gaska | Brüderkrankenhaus St. Josef Paderborn, Klinik f. Hämatologie u. Onkologie, Husenerstr. 46, 33098 Paderborn, GERMANY |
| Gianfranco Giglio | Ospidale A. Cardarelli, Via L. Montalbo snc/Contrada Tappino86100 Campobasso, ITALY |
| Ulrich Graeven | Krankenhaus Maria-Hilf II Franziskushaus, Med. Klinik I, Viersener Str. 450, 41063 Mönchengladbach, GERMANY |
| Jochen Greiner | Diakonie Klinikum Stuttgart, Med. Klinik II, Rosenbergstr. 38, 70176 Stuttgart, GERMANY |
| Frank Griesinger | Pius-Hospital Oldenburg, Klinik für Internistische Onkologie, Georgstr. 12, 26121 Oldenburg, GERMANY |
| Corinna Hahn-Ast | Med. Univ. Poliklinik Bonn, Med. Klinik u. Poliklinik III, Venusberg-Campus 1, 53127 Bonn, GERMANY |
| Mathias Hänel | Krankenhaus Küchwald Chemnitz, Klinik für Innere Medizin III, Bürgerstr. 2, 09009 Chemnitz, GERMANY |
| Frank Hartmann | Klinikum Lippe-Lemgo GmbH, med. Klinik II, Rintelner Str. 85, 32657 Lemgo, GERMANY |
| Jörg Thomas Hartmann | Franziskus-Hospital Bielefeld, Kisker Str. 26, 33615 Bielefeld, GERMANY |
| Holger Hebart | Stauferklinik Schwäbisch Gmünd, Wetzgauer Str. 85, 73557 Mutlangen, GERMANY |
| Michael Heike | Klinikum Dortmund, Medizinische Klinik Mitte, Beurhausstr. 40, 44137 Dortmund, GERMANY |
| Gerhard Heil | Märkische Kliniken GmbH, Klinikum Lüdenscheid, Paulmannshöher Str. 14, 58515 Lüdenscheid, GERMANY |
| Tobias Heintges | Lukaskrankenhaus Neuss, Med. Klinik II, Preußenstr. 84, 41464 Neuss, GERMANY |
| Manfred Hensel | Gemeinschaftspraxis Dres. med. Brust/Schuster/Plöger/Hensel, Q5, 68161 Mannheim, GERMANY |
| Bernd Hertenstein | Klinikum Bremen-Mitte gGmbH, Hämatologie, St.-Jürgen-Str. 1, 28177 Bremen, GERMANY |
| Heinz-Gert Höffkes | Städt. Klinikum Fulda, Med. Klinik III, Pacelliallee 4, 36043 Fulda, GERMANY |
| Martin Hoffmann | Klinikum der Stadt Ludwigshafen, Med. Klinik A, Bremserstr. 79, 67063 Ludwigshafen, GERMANY |
| Jörg Hoffmann | St. Marienkrankenhaus Ludwigshafen, Salzburger Str. 15, 67067 Ludwigshafen, GERMANY |
| Hans-Jürgen Hurtz | GMP Rohrberg/Hurtz/Schmidt/Frank-Gleich, Niemeyerstr. 23, 06110 Halle, GERMANY |
| Elke Jäger | Krankenhaus Nordwest Frankfurt, II. Med. Klinik, Steinbacher Hohl 2-26, 60488 Frankfurt/Main, GERMANY |
| Jan Janssen | Onkologische Praxis Aurich Dres. Reichert/Janssen, Wallinghausenerstraße 8-10, 26603 Aurich, GERMANY |
| Bjarne Anker Jensen | Amtssygehuset i Herlev, Haematologisk afd. L, Herlev Hospital, 2730 Herlev, DENMARK |
| Christoph Kahl | Universität Rostock, Abteilung Hämatologie/Onkologie, Klinik u. Poliklinik für Innere Medizin, Ernst-Heydemann-Str. 6, 18057 Rostock, GERMANY |
| Christoph Kahl | Klinikum Magdeburg, Birkenallee 34, 39130 Magdeburg, GERMANY |
| Ulrich Kaiser | St. Bernward Krankenhaus, Med. Klinik II, Treibestr. 9, 31132 Hildesheim, GERMANY |
| Ulrich Keller | Klinikum rechts der Isar, III. Med. Klinik und Poliklinik, Ismaninger Str. 22, 81675 München, GERMANY |
| Barbara Kempf | Klinikum Landshut, Med. Klinik I, Robert-Koch-Str. 1, 84034 Landshut, GERMANY |
| Alexander Kiani | Klinikum Bayreuth GmbH, Med. Klinik IV, Preuschwitzerstr. 101, 95445 Bayreuth, GERMANY |
| Michael Kiehl | Klinikum Frankfurt (Oder), Abteilung Innere Medizin, Müllroser Chaussee 7, 15236 Frankfurt/Oder, GERMANY |
| Heinz Kirchen | Krankenhaus der Barmherzigen Brüder, I. Med. Abteilung, Nordallee 1, 54292 Trier, GERMANY |
| Bodo Klump | Paracelsus-Krankenhaus Ruit, Klinik für Allgemeine Innere Medizin, Gastroenterologie und Tumormedizin, Kreiskliniken Esslingen, Hedelfinger Str. 166, 73760 Ostfildern |
| Michael Kneba | Städtisches Krankenhaus Kiel, II. Med. Klinik und Poliklinik, Chemnitzstr. 33, 23116 Kiel, GERMANY |
| Yon-Dschun Ko | Johanniter Krankenhaus Bonn, Internistische Onkologie, Johanniterstr. 3-5, 53113 Bonn, GERMANY |
| Georg Köchling | Onkologie Schwarzwald-Alb, Onkologische Schwerpunktpraxis, Albert-Schweitzer-Str. 14, 78052 VS-Villingen, GERMANY |
| H. Köppler | Gemeinschaftspraxis Dres. Köppler/Heymanns/Weide/Thomalla/van Roye, Neversstr. 5, 56068 Koblenz, Germany |
| Detlev Kohl | Ammerland-Klinik GmbH, Lange Str. 38, 26655 Westerstede, GERMANY |
| Beate Krammer-Steiner | Klinikum Rostock Südstadt, Innere Medizin, Südring 81, 18059 Rostock, GERMANY |
| Jürgen Krauter | Städt. Klinikum Braunschweig, Med. Klinik, Celler Str. 38, 38114 Braunschweig, GERMANY |
| Ute Kreibich | Heinrich-Braun-KH/ Städtisches Klinikum Zwickau, Klinik für Innere Medizin II, Karl-Keil-Str. 35, 08009 Zwickau, GERMANY |
| Stephan Kremers | Caritas-Krankenhaus Lebach, Heeresstr. 49, 66822 Lebach, GERMANY |
| Ekkehard Ladda | Onkologische Schwerpunktpraxis, Nürnberger Str. 12, 92318 Neumarkt, GERMANY |
| Christof Lamberti | Klinikum Coburg GmbH, Med. Klinik V, Hämatologie, Intern. Onkologie, Ketschendorfer Str. 33, 96450 Coburg, GERMANY |
| Helmut Lambertz | Klinikum Garmisch-Partenkirchen, Auenstr. 6, 82467 Garmisch-Partenkirchen, GERMANY |
| Elisabeth Lange | Evangelisches Krankenhaus Hamm, Med. Klinik, Hämatologie/Onkologie, Werler Str. 110, 59063 Hamm, GERMANY |
| Eva Lengfelder | Universitätsmedizin Mannheim, III. Med. Universitätsklinik, Theodor-Kutzer-Ufer 1-3, 68167 Mannheim, GERMANY |
| Christian Lerchenmüller | Onkologische Schwerpunktpraxis, Steinfurter Str. 60 B, 48151 Münster, GERMANY |
| Christina Limmroth | Krankenhaus Holweide, Kliniken der Stadt Köln, Med. Klinik, Neufelderstr. 32, 51069 Köln, GERMANY |
| Hans-Walter Lindemann | St.-Josefs -Hospital Hagen, Hämatologie u. Internistische Onkologie, Bergstr. 56, 58095 Hagen, GERMANY |
| Wolf-Dieter Ludwig | Helios Klinikum Berlin Buch/Robert-Rössle-Klinik, Schwanebecker Chaussee 50, 13125 Berlin, GERMANY |
| Ludwig Lutz | Städt. Krankenhaus München-Harlaching, Sanatoriumsplatz 2, Klinik f. Hämatologie, Onkologie u. Palliativmedizin, 81545 München, GERMANY |
| Andreas Mackensen | Universitätsklinik Erlangen-Nürnberg, Maximiliansplatz 2, 91054 Erlangen |
| Rolf Mahlberg | KA Mutterhaus der Borromäerinnen, Med. Abteilung I, Feldstr. 16, 54290 Trier, GERMANY |
| Luisa Mantovani-Löffler | Klinikum "St. Georg", Delitzscherstr. 141, 04129 Leipzig, GERMANY |
| Reinhard Marks | Universitätsklinikum Freiburg, Abt. Innere Medizin I, Hugstetter Str. 55, 76106 Freiburg, GERMANY |
| Felix Marquard | Praxis Dr. Felix Marquard, Neumarkt 1D, 29221 Celle |
| Patrizio Mazza | Ospedale San Giuseppe Moscati, Ematologia, Via per Martina Franca, 74100 Taranto, ITALY |
| Gerald Meckenstock | St. Josef-Hospital Gelsenkirchen, Abt. für Onkologie u. Hämatologie, Rudolf-Bertram-Platz 1, 45699 Gelsenkirchen, GERMANY |
| Hans-Günther Mergenthaler | Bürgerhospital Stuttgart, Tunzhofer Str. 14-16, 70191 Stuttgart, GERMANY |
| Hans-Günther Mergenthaler | Katharinenhospital Stuttgart, Kriegsbergstr. 60, 70174 Stuttgart, GERMANY |
| Francesco Merli | Hematology Azienda USL-IRCCS di Reggio Emilia, 41100 Reggio Emilia, ITALY |
| Bernd Metzner | Klinikum Oldenburg gGmbH, Abt. Hämatologie/Onkologie, Rahel-Straus-Str. 10, 26133 Oldenburg, GERMANY |
| Jörg Mezger | St.-Vincentius-Krankenhäuser Karlsruhe, Med. Klinik A, Südendstr. 32, 76137 Karlsruhe, GERMANY |
| Fortunato Morabito | Azienda Ospedaliera di Cosenza, Via Felize Migliori 1, 87100 Cosenza, ITALY |
| Lothar Müller | Schwerpunktpraxis f. Hämatologie u. Intern. Onkologie, Annenstr. 11, 26789 Leer, GERMANY |
| Caterina Musolino | Policlinico Universitario "G. Martino" , Via Consolare Valeria, 98100 Messina, ITALY |
| Ralph Naumann | St. Marien-Krankenhaus, Kampenstr. 51, 57072 Siegen, GERMANY |
| Andreas Neubauer | Universitätsklinikum Marburg, Klinik für Innere Medizin, Baldingerstraße, 35033 Marburg, GERMANY |
| Godehard Obst | Praxis für Hämatologie und Internistische Onkologie, Hannovesche Str. 2, 30938 Burgwedel, GERMANY |
| Bettina Peuser | Onkolog. Praxis am Diakonissenhaus Leipzig, Fachärztin für Innere Medizin, Georg-Schwarz-Str. 53, 04179 Leipzig, GERMANY |
| Michael Pfreundschuh | Universitätsklinikum des Saarlandes, Innere Medizin I, 66424 Homburg, GERMANY |
| Grabiella Pinotti | Ospedale di circolo e Fondazione Macchi Varese, Viale Luigi Borri 57, 21100 Varese, ITALY |
| Hans-Jörg Pohl | Virngrund-Klinik Ellwangen, Innere Abteilung, Dalkinger Str. 8-12, 73479 Ellwangen |
| Kurt Possinger | Campus Charité Mitte, Medizinische Klinik, Schumannstr. 20/21, 10117 Berlin, GERMANY |
| Otto Prümmer | Klinikum Kempten-Oberallgäu gGmbH, Innere Medizin III, Hämatologie u. Onkologie, Memminger Str. 50-52, 87439 Kempten, GERMANY |
| Aruna Raghavachar | Helios Klinikum Wuppertal, Med. Klinik I, Heusnerstr. 40, 42283 Wuppertal, GERMANY |
| Andreas Rank | Zentralklinikum Augsburg, II. Med. Klinik, Stenglinstr. 2, 86156 Augsburg, GERMANY |
| Tobias Reiber | Praxis Dr. Reiber, Facharzt f. Innere Medizin, Hämatologie, Internist. Onkologie, Schreiberstr. 20, 79098 Freiburg, GERMANY |
| Hans Reinel | Leopoldina-Krankenhaus Schweinfurt, Med. Klinik II, Gustav-Adolf-Str. 8, 97422 Schweinfurt, GERMANY |
| Eva Römer | Klinikum Idar-Oberstein, Dr.-Ottmar-Kohler-Str. 2, 55743 Idar-Oberstein, GERMANY |
| Volker Runde | Wilhelm-Anton-Hospital, Klinik für Innere Medizin, Hämatologie/Intern. Onkologie, Vossheider Str. 214, 47574 Goch, GERMANY |
| Mario Russo | Osp. S. Vincenzo – Taormina, Contrada Sirina, 98039 Taormina, ITALY |
| Matthias Sandmann | Kliniken St. Antonius Wuppertal, Hämatologie/Onkologie, Carnaper Str. 48, 42283 Wuppertal, GERMANY |
| Thomas Schichtl | Med. Versorgungszentrum Weiden, Sölingerstr. 16, 92637 Weiden, GERMANY |
| Frank Schlegel | St.-Antonius-Hospital Eschweiler, Abteilung für Hämatologie u. Onkologie, Dechant-Deckers-Str. 8, 52249 Eschweiler, GERMANY |
| Christian A. Schmidt | Ernst-Moritz-Arndt-Universität, Med. Universitätsklinik C, Hämatologie und Onkologie, Sauerbruchstr., 17487 Greifswald, GERMANY |
| Christian Schmidt | Klinikum Großhadern, Med. Klinik und Poliklinik III, Marchioninistr. 15, 81366 München, GERMANY |
| Rudolf Schmits | Gemeinschaftspraxis Hämat. und Intern. Onkologie, Am Ludwigsberg 78, 66113 Saarbrücken, GERMANY |
| Clemens Schmitt | Charité, Campus Virchow-Klinikum, Med. Klinik, Hämatologie und Onkologie, Augustenburger Platz 1, 13353 Berlin, GERMANY |
| Norbert Schmitz | Asklepios Klinik St. Georg, Hämatologie, Onkologie, Stammzelltransplantationen, Lohmühlenstr. 5, 20099 Hamburg, GERMANY (former address). For current address please see affiliation number 34 |
| Stephan Schmitz | Gemeinschaftspraxis für Onkologie und Hämatologie Köln, Dres. Schmitz/Steinmetz/Gabor, Sachsenring 69, 50677 Köln, GERMANY |
| Jan Schröder | Gemeinschaftspraxis Dres. med. Schröder/Sieg, Kettwiger Str. 62, 45468 Mülheim/Ruhr, GERMANY |
| Wolfgang Schütte | Städt. Krankenhaus Martha-Maria Halle, Innere Medizin II, Röntgenstr. 1, 06120 Halle, GERMANY |
| Dieter Semsek | Onkologische Gemeinschaftspraxis Marschner, Zeiss, Kirste, Semsek, Wirthstr. 11c, 79110 Freiburg, GERMANY |
| Ofer Shpilberg | Rabin Medical Center, Beilinson Hospital, Hematology departement, Jabutinski 95, 49100 Petah-Tiqwa, ISRAEL |
| Martin Sökler | Universitätsklinikum Tübingen, Med. Klinik und Poliklinik, Otfried-Müller-Str. 10, 72076 Tübingen, GERMANY |
| Ulrike Söling | Gemeinschaftspraxis Dres. Siehl/Söling, Goethestr. 47, 34119 Kassel, GERMANY |
| Martina Stauch | Gemeinschaftspraxis Dr. med. Martina Stauch, Niederbronner Str. 2, 96317 Kronach, GERMANY |
| Hjalmar Steinhauer | Carl-Thiem-Klinikum Cottbus, Med. Klinik II, Thiemstr. 111, 03048 Cottbus, GERMANY |
| Eckhard Thiel | Universitätsklinikum Benjamin Franklin, Charité, Med. Klinik III, Hindenburgdamm 30, 12200 Berlin, GERMANY |
| Van Anh Tran Nguyen | Katharinen Hospital UNNA, Innere Klinik I/Gastroenterologie, Obere Husemannstr. 2, 59423 Unna, GERMANY |
| Ralf Ulrich Trappe | Evang. Diakonie-Krankenhaus gGmbH, Med. Klinik II, Gröpelinger Heerstr. 406-408, 28239 Bremen, GERMANY |
| Lorenz Trümper | Georg-August-Universität Göttingen, Hämatologie und Onkologie, Zentrum für Innere Medizin, Robert-Koch-Str. 40, 37075 Göttingen, GERMANY |
| Dirk Tummes | Hämatologische Praxis, Weberstr. 8, 52070 Aachen, GERMANY |
| Daniele Vallisa | Ematologia - OSP. Civile Piacenza, Via Taverna 49, 29100 Piacenza, ITALY |
| Ursula Vehling-Kaiser | Praxis Dr. med. Vehling-Kaiser, Landgasse 132-135, 84028 Landshut, GERMANY |
| Andreas Viardot | Med. Universitätsklinik Ulm, Innere Abteilung III, Albert-Einstein-Allee 23, 89081 Ulm, GERMANY |
| Alexander Wacker | Kreiskliniken Reutlingen, Klinikum am Steinenberg, Steinenbergstr. 31, 72764 Reutlingen, GERMANY |
| Wolfgang Weber | Praxis für Hämatologie/Intern. Onkologie, Melsunger Str. 11, 34576 Homberg Efze, GERMANY |
| Paul Weber | Krankenhaus Siloah Pforzheim, Klinik für Innere Medizin I, Wilferdinger Str. 67, 75179 Pforzheim |
| Georg Weißenborn | Praxis Dr. Georg Weißenborn, Kirchstr. 7, 27239 Twistringen, GERMANY |
| Swen Weßendorf | Städtische Kliniken, Onkologie/Hämatologie, Gastroenterologie und Infektiologie, Hirschlandstr.97, 73730 Esslingen, GERMANY |
| Mathias Witzens-Harig | Ruprecht-Karls-Universität Heidelberg, Med. Klinik, Abt. Innere Medizin, Im Neuenheimer Feld 410, 69120 Heidelberg, GERMANY |
| Hans-Heinrich Wöltjen | Klinikum Minden, Hans-Nolte-Str. 1, 32429 Minden, GERMANY |

Table S2: UNFOLDER Protocol Amendments

|  | Version | Date of version | Date of approval by the Ethics Committee of the Medical Council of Saarland | Date of activation | Changes |
| --- | --- | --- | --- | --- | --- |
| Initial version | 3.1 | 15 September 2004 | 02 October 2004 | - | - |
| Amendment 1 | 3.2 | 27 June 2005 | 18 August 2005 | - | - |
| Supplement | 3.2.1 | 28 September 2005 | 07 October 2005 | 02 January 2006  (start of recruitment) | Change of patient informed consent form |
| Supplement | 3.2.2 | 06 February 2006 | 02 March 2006 | 06 April 2006 | I.th. CNS prophylaxis also for patients with Burkitt and Burkitt-like lymphoma, addition of rate of secondary neoplasia as secondary endpoint, clarification and editorial changes |
| Amendment 2 | 4.0 | 22 November 2012 | 25 February 2013 | 08 May 2013 | Closure of the two treatment arms (R-CHOP-21, R-CHOP-14) without radiotherapy for patients with bulky disease and/or extranodal involvement, abandonement of i.th. CNS prophylaxis, introduction of prophylactic irradiation of contralateral testis in case of testicular lymphoma, clarification and editorial changes |

Table S3: Baseline patient demographic and disease characteristics of PMBCL and all Non-PMBCL patients in the UNFOLDER trial

|  | PMBCL  (n=136) | | Non-PMBCL  (n=559) | | UNFOLDER trial  total  (n=695) | |
| --- | --- | --- | --- | --- | --- | --- |
| Male | 62 | (46%) | 341 | (61%) | 403 | (58%) |
| Female | 74 | (54%) | 218 | (39%) | 292 | (42%) |
| Age, median (range) | 34 | (18, 60) | 49 | (18, 60) | 47 | (18, 60) |
| LDH > than upper limit of normal | 108 | (79%) | 185 | (33%) | 293 | (42%) |
| Eastern Cooperative Oncology Group Performance status > 1 | 0 | (0%) | 3 | (1%) | 3 | (0.4%) |
| Stage III/ IV | 10 | (7%) | 286 | (51%) | 296 | (43%) |
| Age-adjusted International Prognostic Index |  |  |  |  |  |  |
| 0  1  2 | 20  114  2 | (15%)  (84%)  (2%) | 91  462  6 | (16%)  (83%)  (1%) | 111  576  8 | (16%)  (83%)  (1%) |
| Stage I  II  III  IV | 63  63  2  8 | (46%)  (46%)  (2%)  (6%) | 96  177  122  164 | (17%)  (32%)  (22%)  (29%) | 159  240  124  172 | (23%)  (35%)  (18%)  (25%) |
| Extralymphatic involvement | 31 | (23%) | 293 | (52%) | 324 | (47%) |
| Extralymphathic involvement > 1 | 4 | (3%) | 109 | (20%) | 113 | (16%) |
| Bulk ≥ 7.5 cm | 131 | (96%) | 265 | (47%) | 396 | (57%) |
| B symptoms | 48 | (35%) | 104* | (19%) | 152* | (22%) |
| Bone marrow involvement | 0 | (0%) | 41 | (7%) | 41 | (6%) |

*4 missing values

Table S4: Sites of extralymphatic involvement in PMBCL patients

| Localisation | R-CHOP-21  (n=27) | | R-CHOP-14  (n=22) | | R-CHOP-21 +radiotherapy  (n=43) | | R-CHOP-14 +radiotherapy  (n=39) | | all PMBCL  (n=131) | |
| --- | --- | --- | --- | --- | --- | --- | --- | --- | --- | --- |
| Bone marrow | 0 | (0%) | 0 | (0%) | 0 | (0%) | 0 | (0%) | 0 | (0%) |
| Lung | 0 | (0%) | 2 | (9%) | 5 | (12%) | 2 | (5%) | 9 | (7%) |
| Liver | 0 | (0%) | 0 | (0%) | 0 | (0%) | 0 | (0%) | 0 | (0%) |
| Skeletal | 0 | (0%) | 0 | (0%) | 0 | (0%) | 0 | (0%) | 0 | (0%) |
| Pleura | 0 | (0%) | 1 | (4%) | 4 | (9%) | 1 | (3%) | 6 | (5%) |
| Pericard | 1 | (4%) | 0 | (0%) | 5 | (12%) | 3 | (8%) | 9 | (7%) |
| CNS | 0 | (0%) | 0 | (0%) | 0 | (0%) | 0 | (0%) | 0 | (0%) |
| Stomach | 0 | (0%) | 0 | (0%) | 0 | (0%) | 0 | (0%) | 0 | (0%) |
| Small intestine | 0 | (0%) | 0 | (0%) | 0 | (0%) | 0 | (0%) | 0 | (0%) |
| Colon | 0 | (0%) | 0 | (0%) | 0 | (0%) | 0 | (0%) | 0 | (0%) |
| Orbita | 0 | (0%) | 0 | (0%) | 0 | (0%) | 0 | (0%) | 0 | (0%) |
| Paranasal sinuses | 0 | (0%) | 0 | (0%) | 0 | (0%) | 0 | (0%) | 0 | (0%) |
| Main nasal cavity | 0 | (0%) | 0 | (0%) | 0 | (0%) | 0 | (0%) | 0 | (0%) |
| Mouth region | 0 | (0%) | 0 | (0%) | 0 | (0%) | 0 | (0%) | 0 | (0%) |
| Tongue | 0 | (0%) | 0 | (0%) | 0 | (0%) | 0 | (0%) | 0 | (0%) |
| Salivary glands | 0 | (0%) | 0 | (0%) | 0 | (0%) | 0 | (0%) | 0 | (0%) |
| Thyroid gland | 0 | (0%) | 1 | (4%) | 0 | (0%) | 1 | (3%) | 2 | (2%) |
| Mammary gland | 0 | (0%) | 0 | (0%) | 0 | (0%) | 0 | (0%) | 0 | (0%) |
| Peritoneum | 0 | (0%) | 0 | (0%) | 0 | (0%) | 0 | (0%) | 0 | (0%) |
| Pancreas | 0 | (0%) | 0 | (0%) | 0 | (0%) | 0 | (0%) | 0 | (0%) |
| Kidney | 0 | (0%) | 0 | (0%) | 0 | (0%) | 0 | (0%) | 0 | (0%) |
| Adrenal gland | 0 | (0%) | 0 | (0%) | 0 | (0%) | 0 | (0%) | 0 | (0%) |
| Urinary bladder | 0 | (0%) | 0 | (0%) | 0 | (0%) | 0 | (0%) | 0 | (0%) |
| Testes | 0 | (0%) | 0 | (0%) | 0 | (0%) | 0 | (0%) | 0 | (0%) |
| Ovary | 0 | (0%) | 0 | (0%) | 0 | (0%) | 0 | (0%) | 0 | (0%) |
| Uterus | 0 | (0%) | 0 | (0%) | 0 | (0%) | 0 | (0%) | 0 | (0%) |
| Skin | 0 | (0%) | 0 | (0%) | 0 | (0%) | 0 | (0%) | 0 | (0%) |
| Soft tissues | 3 | (11%) | 0 | (0%) | 1 | (2%) | 4 | (10%) | 8 | (6%) |
| Ascites | 0 | (0%) | 0 | (0%) | 0 | (0%) | 0 | (0%) | 0 | (0%) |
| Other* | 1 | (4%) | 0 | (0%) | 0 | (0%) | 0 | (0%) | 1 | (1%) |

*Vena cava superior

Table S5: Causes of death in PMBCL

|  | R-CHOP-21  (n=27) | |  | R-CHOP-14  (n=22) | | R-CHOP-21 +radiotherapy  (n=43) | | R-CHOP-14 +radiotherapy  (n=39) | | all PMBCL  (n=131) | |
| --- | --- | --- | --- | --- | --- | --- | --- | --- | --- | --- | --- |
| Lymphoma related | 1 | |  | 1 | | 0 | | 1 | | 3 | |
| Concomitant diseases | 0 | |  | 0 | | 1 | | 0 | | 1 | |
| Total  (patients died) | 1 | (4%) |  | 1 | (5%) | 1 | (2%) | 1 | (3%) | 4 | (3%) |

Table S6: Rates of complete response, progressive disease, and relapse rates in PMBCL

|  | R-CHOP-21  (n=27) | | R-CHOP-14  (n=22) | | R-CHOP-21 +radiotherapy  (n=43) | | R-CHOP-14 +radiotherapy  (n=39) | | all PMBCL  (n=131) | |
| --- | --- | --- | --- | --- | --- | --- | --- | --- | --- | --- |
| complete response rates | 22/27 | (82%) | 19/22 | (86%) | 41/43 | (95%) | 36/39 | (92%) | 118/131 | (90%) |
| 95% CI | (63%; 94%) | | (65%; 97%) | | (84%; 99%) | | (79%; 98%) | | (84%; 95%) | |
| progressive disease rates | 1/27 | (4%) | 1/22 | (4%) | 1/43 | (2%) | 1/39 | (3%) | 4/131 | (3%) |
| 95% CI | (0.1%; 19%) | | (0.1%; 22%) | | (0.03%; 12%) | | (0.1%; 14%) | | (1%; 8%) | |
| relapse rates | 3/22 | (14%) | 0/19 | (0%) | 0/41 | (0%) | 0/36 | (0%) | 3/118 | (2%) |
| 95% CI | (3%; 35%) | | (0%; 18%) | | (0%; 9%) | | (0%; 10%) | | (0·3%; 6%) | |

Table S7: Toxicity of chemoimmunotherapy per patient in PMBCL

| CTC grade 3/4 | R-CHOP-21  (n=27) | | R-CHOP-14  (n=22) | | R-CHOP-21 +radiotherapy  (n=43) | | R-CHOP-14 +radiotherapy  (n=39) | | all PMBCL  (n=131) | |
| --- | --- | --- | --- | --- | --- | --- | --- | --- | --- | --- |
| Leukocytopenia* | 10/16 | (62%) | 4/8 | (50%) | 13/20 | (65%) | 10/22 | (45%) | 37/66 | (56%) |
| Thrombocytopenia | 0/27 | (0%) | 0/21 | (0%) | 0/42 | (0%) | 0/35 | (0%) | 0/125 | (0%) |
| Anaemia | 0/27 | (0%) | 0/21 | (0%) | 3/41 | (7%) | 1/34 | (3%) | 4/123 | (3%) |
| Nausea | 1/24 | (4%) | 1/19 | (5%) | 2/43 | (5%) | 2/38 | (5%) | 6/124 | (5%) |
| Vomiting | 1/24 | (4%) | 1/19 | (5%) | 1/43 | (2%) | 0/38 | (0%) | 3/124 | (2%) |
| Diarrhoea | 0/25 | (0%) | 0/19 | (0%) | 0/43 | (0%) | 1/38 | (3%) | 1/125 | (1%) |
| Constipation | 0/24 | (0%) | 0/19 | (0%) | 0/43 | (0%) | 0/38 | (0%) | 0/124 | (0%) |
| Mucous membranes/ Mucositis | 0/25 | (0%) | 0/19 | (0%) | 0/43 | (0%) | 0/38 | (0%) | 0/125 | (0%) |
| Arrhythmia | 0/24 | (0%) | 0/19 | (0%) | 0/43 | (0%) | 2/38 | (5%) | 2/124 | (2%) |
| Cardiac functions | 0/23 | (0%) | 0/19 | (0%) | 0/43 | (0%) | 1/38 | (3%) | 1/123 | (1%) |
| Haematuria | 0/24 | (0%) | 0/19 | (0%) | 0/42 | (0%) | 0/38 | (0%) | 0/123 | (0%) |
| Haemorrhagic Cystitis | 0/24 | (0%) | 0/19 | (0%) | 0/42 | (0%) | 0/38 | (0%) | 0/123 | (0%) |
| Sensory | 0/24 | (0%) | 1/19 | (5%) | 4/43 | (9%) | 0/36 | (0%) | 5/122 | (4%) |
| Mood | 0/24 | (0%) | 0/19 | (0%) | 0/43 | (0%) | 0/38 | (0%) | 0/124 | (0%) |
| Allergy | 0/24 | (0%) | 0/19 | (0%) | 0/43 | (0%) | 0/38 | (0%) | 0/124 | (0%) |
| Infection | 0/26 | (0%) | 4/21 | (19%) | 6/43 | (14%) | 4/38 | (10%) | 14/128 | (11%) |

*based on blood counts within nadir interval day 11-14 (R-CHOP-21) and day 8-10 (R-CHOP-14)

Table S8: 3-year event-free, progression-free, and overall survival in PMBCL

|  | R-CHOP-21  (n=27) | R-CHOP-14  (n=22) | R-CHOP-21 +radiotherapy  (n=43) | R-CHOP-14 +radiotherapy  (n=39) | all PMBCL  (n=131) |
| --- | --- | --- | --- | --- | --- |
| 3-year event-free survival | 70% | 86% | 95% | 92% | 88% |
| 95% CI | (53%; 88%) | (72%; 100%) | (89%; 100%) | (84%; 100%) | (82%; 93%) |
| 3-year progression-free survival | 85% | 96% | 95% | 95% | 93% |
| 95% CI | (72%; 98%) | (87%; 100%) | (89%; 100%) | (88%; 100%) | (89%; 97%) |
| 3-year overall survival | 96% | 96% | 98% | 97% | 97% |
| 95% CI | (89%; 100%) | (87%; 100%) | (93%; 100%) | (92%; 100%) | (94%; 100%) |

Table S9: Acute Toxicity of radiotherapy in PMBCL

| CTC grade 3/4 | R-CHOP-21 +radiotherapy  (n=39*) | | R-CHOP-14 +radiotherapy  (n=37*) | | all PMBCL  (n=76*) | |
| --- | --- | --- | --- | --- | --- | --- |
| Haemoglobin | 0/29 | (0%) | 0/28 | (0%) | 0/57 | (0%) |
| Leucocytes | 3/29 | (10%) | 1/28 | (4%) | 4/57 | (7%) |
| Platelets | 0/29 | (0%) | 1/28 | (4%) | 1/57 | (2%) |
| Nausea | 0/32 | (0%) | 1/30 | (3%) | 1/62 | (2%) |
| Vomiting | 0/33 | (0%) | 0/30 | (0%) | 0/63 | (0%) |
| Diarrhoea | 0/33 | (0%) | 0/30 | (0%) | 0/63 | (0%) |
| Oesophagitis/ Dysphagia | 2/35 | (6%) | 2/33 | (6%) | 4/68 | (6%) |
| Constipation | 0/33 | (0%) | 0/29 | (0%) | 0/62 | (0%) |
| Mucous membranes/ Mucositis | 0/33 | (0%) | 0/30 | (0%) | 0/63 | (0%) |
| Salivary glands | 0/33 | (0%) | 0/30 | (0%) | 0/63 | (0%) |
| Arrhythmia | 0/31 | (0%) | 0/29 | (0%) | 0/60 | (0%) |
| Cardiac function | 0/31 | (0%) | 0/28 | (0%) | 0/59 | (0%) |
| Dyspnoea | 0/34 | (0%) | 0/30 | (0%) | 0/64 | (0%) |
| Larynx | 0/29 | (0%) | 1/30 | (3%) | 1/59 | (2%) |
| Hematuria | 0/28 | (0%) | 0/28 | (0%) | 0/56 | (0%) |
| Sensory | 0/28 | (0%) | 0/27 | (0%) | 0/55 | (0%) |
| Mood | 0/28 | (0%) | 0/29 | (0%) | 0/57 | (0%) |
| Otitis | 0/28 | (0%) | 0/28 | (0%) | 0/56 | (0%) |
| Keratitis | 0/28 | (0%) | 0/28 | (0%) | 0/56 | (0%) |
| Nose/ sense of smell | 0/28 | (0%) | 0/28 | (0%) | 0/56 | (0%) |
| Skin/ subcutis local | 1/28 | (4%) | 0/27 | (0%) | 1/55 | (2%) |
| Infection | 1/27 | (4%) | 0/29 | (0%) | 1/56 | (2%) |

* Patients who received radiotherapy


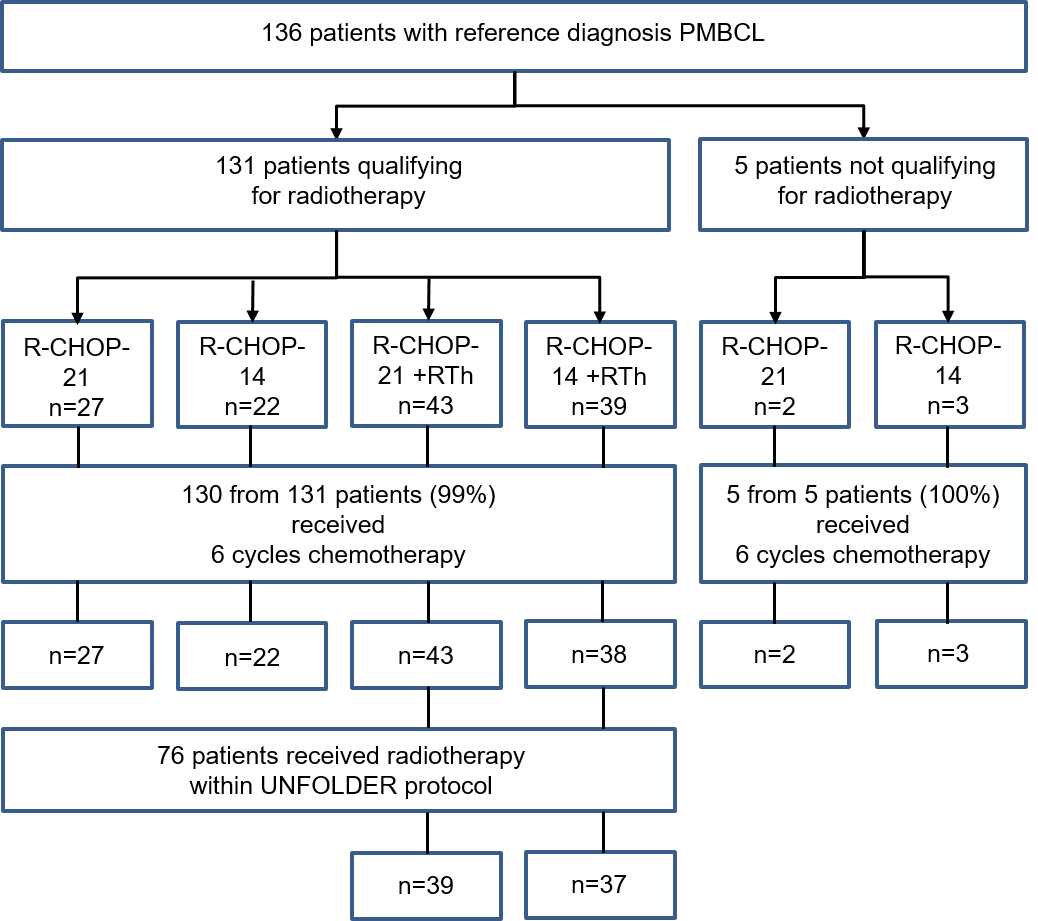


**Supplementary Figure S1: CONSORT diagram.** Diagram showing PMBCL subgroup within the UNFOLDER trial.


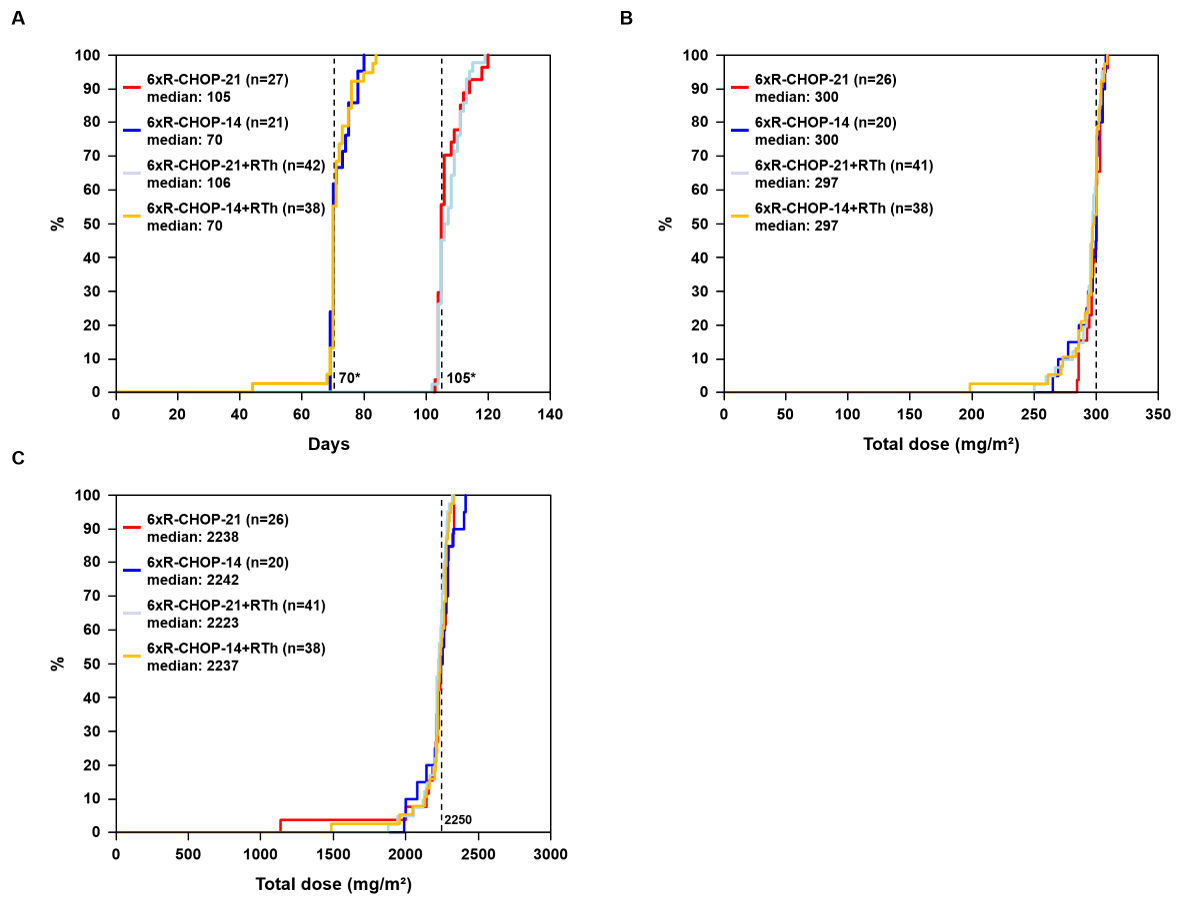


**Supplementary Figure S2: Total duration of CHOP chemotherapy, absolute dose of doxorubicine and rituximab.** Graphs show total duration of CHOP chemotherapy depicted by the number of days from first day of the first cycle until the first day of the sixth cycle [* depicts the planned duration] (A, n=128) and absolute dose of doxorubicine (B, n=125), and rituximab (C, n=125) according to treatment arm for all PMBCL patients (n=131).


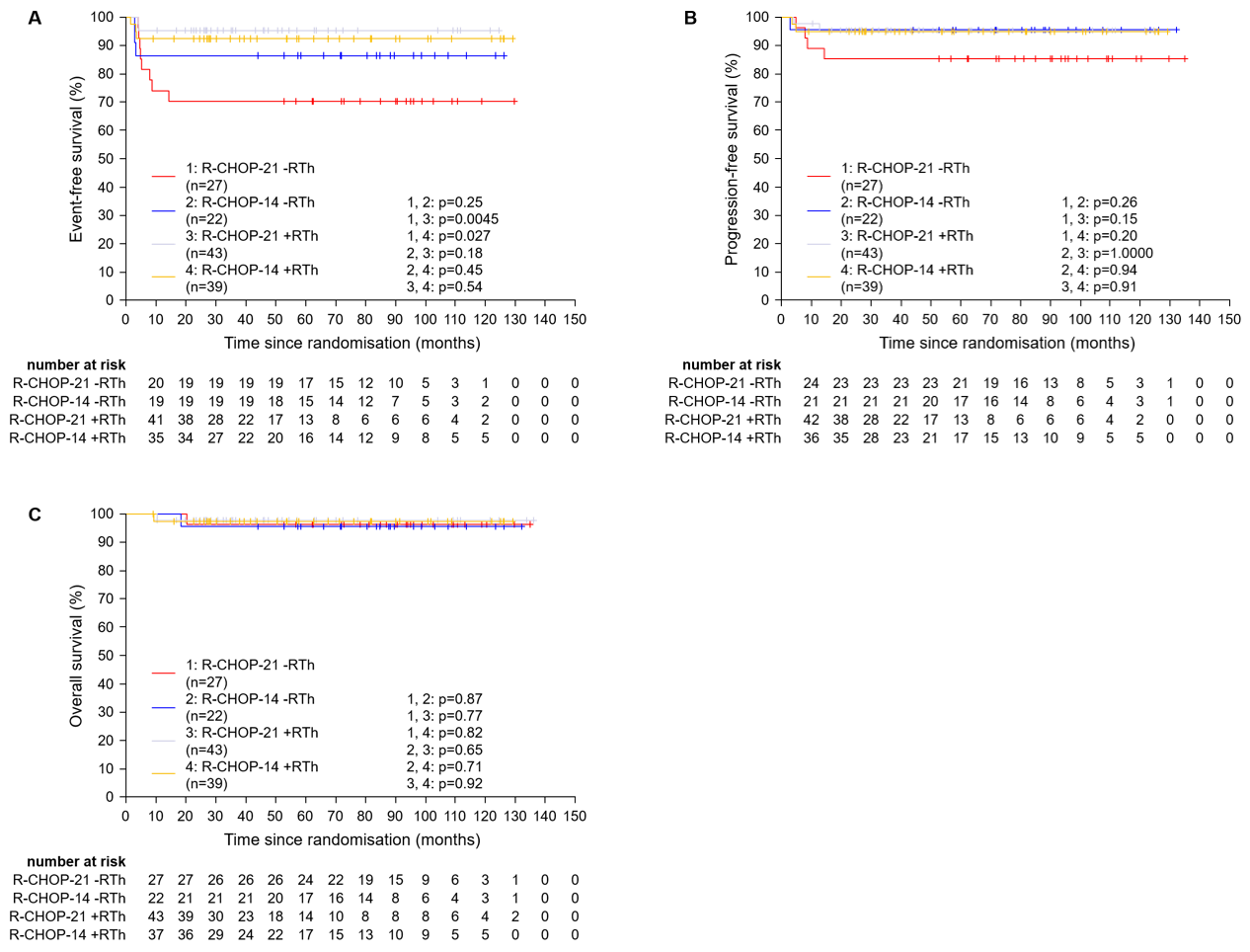


**Supplementary Figure S3: Event- free, progression-free, and overall survival according to treatment arms.** Graphs show EFS (A), PFS (B) and OS (C) for all PMBCL patients (n=131).


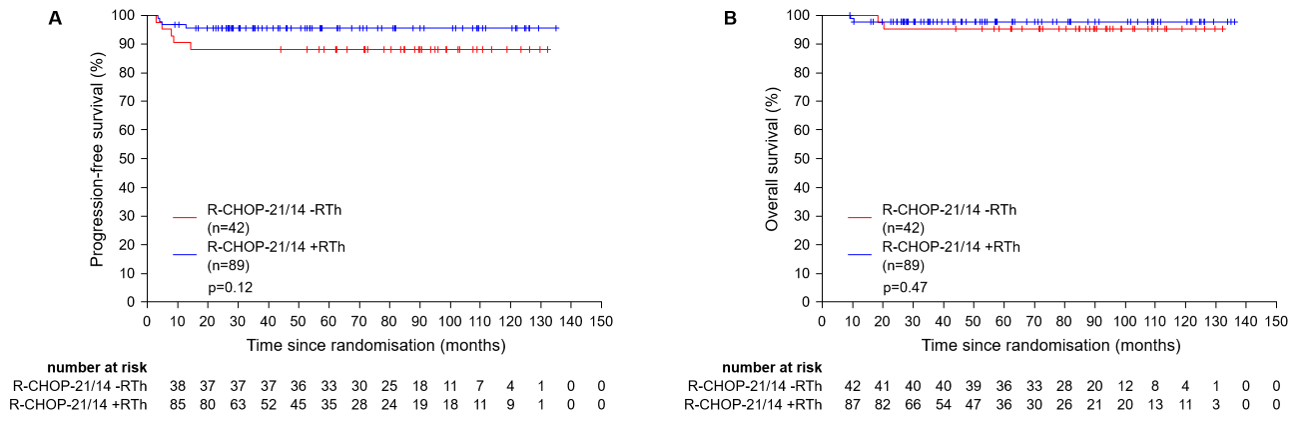


**Supplementary Figure S4: Progression-free and overall survival according to treatment given in radiotherapy- or observation-arm [radiotherapy vs. observation] for all PMBCL patients.** Graphs show PFS (A) and OS (B), (n=131).
